# The effects of exercise on neuromuscular function in people with chronic neck pain: A systematic review and meta-analysis

**DOI:** 10.1371/journal.pone.0315817

**Published:** 2024-12-19

**Authors:** Angelo Marco Dirito, Deepa Abichandani, Ferozkhan Jadhakhan, Deborah Falla

**Affiliations:** 1 Centre of Precision Rehabilitation for Spinal Pain (CPR Spine), School of Sport, Exercise and Rehabilitation Sciences, University of Birmingham, Birmingham, United Kingdom; 2 Department of Physiotherapy, London South Bank University, London, United Kingdom; 3 Faculty of Health, Education and Life Sciences, Birmingham City University, Birmingham, United Kingdom; Lahore University of Biological and Applied Sciences, PAKISTAN

## Abstract

**Background:**

Differences in cervical neuromuscular function are commonly observed between people with and without chronic neck pain. Exercise may improve cervical neuromuscular function of people with neck pain although the evidence for this has not been systematically reviewed.

**Objective:**

To systematically review the existing evidence on the effect of exercises targeting the neck muscles on neuromuscular function in people with chronic non-specific neck pain.

**Methods:**

This systematic review was conducted based on a registered protocol (CRD42021298831) with searches conducted on the following databases from inception to 21st October 2023: MEDLINE, CINAHL, Web of Science, Scopus, AMED, Google Scholar, Open Grey and Zetoc. Studies of interest were trials investigating neuromuscular adaptations to a program of exercise targeting the neck muscles (>2 weeks) in people with chronic non-specific neck pain. Two reviewers independently screened the studies and performed data extraction, risk of bias assessment, and rated the overall certainty of the evidence (GRADE).

**Results:**

Fourteen articles from 2110 citations were included. There is moderate certainty of evidence that the use of craniocervical flexion training (either in isolation or in combination with resistance training) can induce neural adaptations within the neck muscles. A meta-analysis showed a reduction in sternocleidomastoid muscle activity after neck exercise interventions compared to control interventions.

**Conclusion:**

The articles included in this systematic review confirmed that exercise can result in neuromuscular adaptations within neck muscles, as measured by electromyography. Specificity of training was seen to be relevant for the type of neuromuscular adaptations induced.

## Introduction

Neck pain is the third leading cause of years lived with disability [1] and is associated with substantial financial and social burden [2]. Neck pain affects all age groups and genders, with the greatest incidence observed for people aged between 45–54 years and for women [3]. Overall, in 2017 the global age-standardized prevalence and incidence rate of neck pain were 3551.1 and 806.6 per 100,000 population respectively [3].

Physical therapy is considered the first line treatment for musculoskeletal disorders with evidence particularly supporting the role of exercise for managing pain, range of motion and disability in people with chronic neck pain [4]. Moreover, some studies emphasize that exercise can be used to enhance neuromuscular function of people with neck pain, potentially leading to better long-term outcomes [5]. This is relevant given the extensive literature documenting changes in neuromuscular function of the neck in people with chronic neck pain [6–8]. This includes increased muscle co-activation [9], changes in coordination between the deep and superficial flexor muscles [10], reduced specificity of neck muscle activity [11] and delayed onset of neck muscles in response to perturbations [12, 13]. People with neck pain also commonly present with reduced neck muscle strength [9, 14] and less endurance [15, 16].

Several systematic reviews have investigated the effect of exercise on neck pain [4, 17, 18]. Overall, these systematic reviews have focused mainly on patient reported outcome measures such as changes in pain intensity, disability, quality of life, global perceived effect, and patient satisfaction. To the best of our knowledge, only one systematic review has investigated the effect of neck exercise on neuromuscular function in people with neck pain [19]. In this review, Blomgren et al. [19] specifically analyzed the effects of craniocervical flexion (CCF) training compared with other forms of training or no exercise on cervical neuromuscular function, in addition to neck muscle size, kinematics, and kinetics. Although this is of relevance, there are many other forms of neck exercise which are relevant to consider when examining the influence of exercise on neuromuscular function, such as resistance training. Thus, in the current systematic review, we consider the effects of all forms of exercise targeting neck muscles and their effect on neuromuscular function in patients with chronic non-specific neck pain. Such knowledge may assist clinicians when planning effective exercise interventions to address neuromuscular function for their patients.

The primary aim of this systematic review is to synthesise the current literature on the effect of exercise targeting the neck muscles performed for at least two weeks on neuromuscular function (e.g., neck muscle strength, endurance, and muscle activity) in people with chronic non-specific neck pain. A secondary objective is to observe if changes in pain intensity and disability are concordant with any physiological changes induced by exercise. This systematic review is based on two hypotheses: 1. exercise may enhance cervical neuromuscular function in people with chronic neck pain and 2. changes in neuromuscular function may explain the positive influence of exercise on pain and disability in people with chronic neck pain.

## Methods

### Protocol and registration

This systematic review was registered on PROSPERO as protocol No. CRD42021298831 and is reported according to the Preferred Reporting Items for Systematic Review and Meta-Analyses (PRISMA) [20] [S1 File]. The review is based on the updated method guidelines for systematic reviews from the Cochrane back and neck group [21].

### Eligibility criteria

Eligibility criteria were based on the PICOS (Population, intervention, comparator, outcome, and study design) framework [22].

#### Population

Studies of adults aged ≥ 18 years with chronic non-specific neck pain. Any study where the population was not defined as non-specific neck pain were excluded. This includes studies where participants have neck pain due to pathologies including degenerative diseases, tumors or inflammatory rheumatic disorders and neck pain attributed to an injury (e.g., whiplash). Studies on mechanical neck pain were also excluded as it was not clear from these studies whether a specific pathoanatomical source of pain had been identified.

#### Intervention

Any form of exercise targeting the neck region (e.g., motor control, strengthening, endurance) performed for a minimum of 2 weeks without any other additional treatment besides advice or education. This cutoff for training duration was chosen to allow sufficient time for neural adaptations to occur [23]. Trials of rehabilitation or physiotherapy interventions with no specific reference to exercise were excluded.

#### Comparator

Comparator studies included no intervention or passive interventions (e.g., manual therapy, education only) or general practitioner management.

#### Outcome measures

Outcomes included those measured with electromyography (EMG) such as the amplitude of muscle activity, timing of muscle activity and measures of muscle fatigability. Measures of corticospinal excitability assessed via transcranial magnetic stimulation were also considered. Outcomes which were measures of motor output such as muscle strength, rate of torque development, and endurance were also included. Studies focusing only on muscle morphology e.g., muscle size or fatty tissue, were excluded. Secondary outcomes were self-reported measures of pain intensity and disability.

#### Study design

Randomized controlled trials (RCTs), controlled clinical trials and non-randomised studies of exercise interventions.

#### Exclusion criteria

Any study not written in English, studies not yet completed and studies that had been published only as a conference abstract or thesis were excluded. Other languages were excluded due to limited resources to translate. No publication time restriction was set for this systematic review.

### Information sources

The electronic databases that were searched from inception until the 21st of October 2023 included MEDLINE, CINAHL, Web of Science, Scopus, AMED, Google Scholar, Open Grey and Zetoc.

### Search strategy

A comprehensive search for MEDLINE was conducted. The search strategy was generated with Ovid and subsequently the key words of the search strategy were modified using truncation and wildcard searches for the databases listed above [S2 File].

### Study selection

All records retrieved in the database search were imported into Endnote (Clarivate Analytics, USA) publication management software. Titles and abstracts were screened, independently by two reviewers (AMD/DA), according to the eligibility criteria. Disagreements were resolved by discussion between the two reviewers. A third reviewer (DF) was available in case of further disagreement [S3 File]. In the second stage of screening, the two reviewers examined the full text [S4 File] to determine their final eligibility [S5 File].

### Data extraction process and data items

Two reviewers (AMD and DA) extracted data independently from the articles included in the review; they then compared data extracted and created a single file. Data extracted from the articles included, study details (author, date, location), sample size, participant information, outcome measures, follow up periods (Table 1). Moreover, a description of the intervention was obtained using the Template for Intervention Description and Replication (TIDieR) checklist as guidance (Table 2) [24].

**Table 1:** Study characteristics according to the TIDieR checklist.

**Study**			**Study Characteristics**					**Participant Information**				
**Author**	**Year**	**Country**	**Type**	**Power calculation**	**Adherence**	**Treatment Fidelity**		**Description**	**Age (mean ± SD)**	**Sample size (%F)**	**Inclusion criteria**	
**Beer**	**2012**	Australia	Preliminary RCT	Not Reported	Data from the exercise diaries indicated that subjects performed the exercise, on average, 15.1 ±3.8 times per day (range 8.1–20.4 times per day) over the 2 week intervention period	Patients had one supervised session, they had a diary to record the number of time they performed the exercise. Twice per week they were assessed to ensure correct performance of the exercise		General population	ExGp. 26,8 ± 9,6; CGp.31,7 ± 13,3	20 (50%)	age range 18–54 years, persistent neck pain, poor performance in CCFT	
**Borisut**	**2013**	Thailand	RCT	Not Reported	Exercise compliance in this study was over 80% in all groups	Patients kept a logbook for monitoring		Computer workers	1Gp. 32,72 ± 3,11; 2Gp.30,4 ± 3,54; 3Gp. 30,16 ± 2,96; CGp. 29,32 ± 3,11	100 (100%)	age range: 20–35 years, history of intermittent work-related neck pain lasting for more than 6 months, worked with a computer at least 4 hours each working day, pain level 30mm on 100mm VAS	
**Falla**	**2006**	Australia	Randomised trial	Reported (difference in rate of change of the EMG mean frequency between a group of neck pain and asymptomatic subjects) 26 subject per group	100%	Endurance-strength training group: 91.0 ± 0.12%; Cranio-cervical flexion training group: 94.8 ± 0.06%		Female with a history of chronic neck pain >3 months	Endurance-strength training group: 37.7 ± 9.9; Cranio-cervical flexion training group: 38.1 ±10.7	58 (100%)	NDI ≤15%	
**Falla**	**2008**	Australia	Randomised trial	Not reported	100%	100%		Female with a history of chronic neck pain of greater than 3-month duration	Endurance-strength training group: 38.1 ±10.7; Cranio-cervical flexion training group: 37.7 ± 10.1	57 (100%)	NDI <15, no cervical spine surgery, no neurological signs, not have been enrolled in a neck exercise programme in the past 12 months	
**Falla**	**2013**	Germany	RCT	Reported (NDI based), 23 subjects per group	Not Declared	Patients received personal instruction and supervision by a physiotherapist for ∼30 min once per week for 8 weeks of trial.		General population	ExGp. 39,1 ± 8,7; CGp. 38,6 ± 9	46 (100%)	age range: 18–50 years, suffering from persistent neck pain and disability limiting their daily physical activity for at least 1 year	
**Mendes-Fernandes**	**2023**	Portugal	RCT	Reported (NDI based)	50/52	100%		Women with non-specific neck pain from the community of Guarda County, Portugal	Postural Gp. 47.84 ± 8.86; Neck SE Gp. 53.80 ± 7.74	50 (100%)	Women aged between 30 and 65 years with chronic non- specific neck pain lasting for at least 12 weeks, with pain intensity greater or equal to 2 in the numerical pain rating Scale	
**Ghaderi**	**2017**	Iran	RCT	Not reported	Not reported	Not reported		Participants from Physiotherapy clinic of Rehabilitation Faculty of Tabriz University of Medical Sciences	ExGp. 35.97 ± 2.5;; CGp. 36.34 ± 3.06	40 (Not declared)	Chronic neck pain without neurologic signs for more than 12 weeks, severity of pain not more than 6.5 (VAS), right hand dominancy, no surgery/fracture/trauma/fibromyalgia/disc herniation/deformities in neck or shoulder, no treatments for neck or shoulder, no pregnancy	
**Javdaneh**	**2020**	Iran	RCT	Based on VAS score	Not reported	100%		Outpatients	Scapular exercise group: 29 ± 4.37; Combined (exercise+cognitive) group: 30 ± 6.01; Control group: 28 ± 4.77	72 (47.2%)	Age between 20 and 45 years, suffering from ongoing bilateral neck pain for at least 3 months, moderate pain intensity (30–70 VAS), 37 or above Tampa scale, having cognitive-behavioral problems, be able to perform pain-free shoulder abduction to at least 160°, bilateral scapular downward rotation, no severe psychopathology, no neck or shoulder surgery, no shoulder pain, no intervention for neck-shoulder pain	
**Jull**	2009	Australia	Randomised trial	80% power, and 95% confidence based on EMG	93.5%	100%		Female subject with chronic neck pain greater than 3 months duration	Cranio-cervical flexion training group: 39.6 ± 12.2; Strength training group: 37.1 ± 10.3	46 (100%)	Non-severe neck symptoms (NDI <15/50), poor performance in the CCFT, unable to control more than the second stage of the test, no cervical spine surgery, no neurological signs, no neck exercise program in the past 12 month.	
**Lidegaard**	2013	Denmark	RCT	Not reported	Training group: 86.8%; Control group: 89%	93.4%		Employees from one large office workplace characterized by computer for the majority of working time	Training group: 41.7 ± 10.8; Control group: 40.5 ± 7.27	30 (100%)	No cardiovascular or cerebrovascular accident, no fibromyalgia, no rheumatoid arthritis, no cervical disc prolapse, no whiplash, no serious traumatic injury of the neck or shoulder, no chronic disease, no pregnancy, no more than 2h pf vigorous physical exercise per week	
**Lundblad**	1999	Sweden	RCT	Not Reported	Not Reported	Not Reported		Female workers	PTGp. 33 ± 9; FGp. 35 ± 1; CGp. 34 ± 9	97 (100%)	Neck shoulder complaints	
**Ma**	2011	China	RCT	80% power and 5% type I error (based on VAS and NDI)	16.6% drop-out	most participants reported doing the exercises for 6 to 7d/wk.		General population	33,3 ± 9.7; Grp. A (biofeedback): 31.3±8.6; Grp. B (active exercise): 34.2±10.3; Grp. C (passive treatment): 35.3±9.4; Grp. D (control): 30.0±10.3	60 (66%)	Daily computer user, past and present history of computer-related neck and shoulder discomfort, worked on a computer for at least 5 years, no more than 3 months out of work during the previous 5 years, neck and/or shoulder pain on at least 30 days during the previous year, experienced neck and/or shoulder pain in the previous 7 days, no pain in more than 3 body regions, no arthritis or joint disorders, no neck and/or shoulder pain on fewer than 8 days during the previous 1 year, no taking muscle relaxants, no tumors or inflammatory diseases.	
**Mehri**	2020	Iran	RCT	Reported (NDI based). 12 subjects per group	Not Reported	The exercise program was performed under the supervision of the physiotherapist and corrective exercise trainer		General population	ExGp. 37,76 ± 3,83; CGp. 35,54 ± 5,4	32 (100%)	Age range: 30–40 years, pain intensity in the neck/shoulder of at least 3 on a 0 to 9 scale, neck pain for more than 3 months, right-handed dominance, at least 2 deficits in neck motion control according to a reliable test battery, cervical and shouder angles >50° and >52°	
**Yan**	2022	China	RCT	Not Reported	Not Reported	Not Reported		General population	ExGp. 57,86 ± 3,16; CGp. 58,31 ± 4,44	25 (100%)	1) Female patients with chronic neck pain, age range from 40–65 years; 2) The presence of neck and shoulder discomfort, pain, limited movement, and other symptoms for more than 6 months; 3) Cervical muscle tension, neck and shoulder pressure points were found by physical examination	
**Study**			**Intervention Information**					**Control information**				
**Author**	**Year**	**Country**	**Name**	**Materials**	**What**	**When and how much**	**Tailoring and modification**	**Name**	**Materials**	**What**	**When and how much**	**Tailoring and modification**
**Beer**	**2012**	Australia	Postural exercise intervention		gently "lift the base of the skull from the top of the neck as if to lengthen the cervical spine	2 week, holding the position for 10s ideally every 15/20 min throughout their waking day		Control group		no treatment		
**Borisut**	**2013**	Thailand	Strength-endurance Exercise Group	weights	neck flexion and extension in lying position	12 weeks (first phase: 4 week, second phase: 8 weeks)	first phase 12–15 rep of a weight they could lift 12 times, second phase 3 set per 15 repetitions with 1 min rest.	Control group		no treatment		
			Craniocervical-flexion exercise Group	Chattanooga Pressure Biofeedback Unit (Stabilizer)	supine slow head flexion from 20mmHg to 22-30mmHg maintaining it for 10sec for 15rep, 10sec rest each rep	12 weeks						
			Combined exercise Group	weights and Chattanooga Pressure Biofeedback Unit (Stabilizer)	the combination of Craniocervical-flexion exercise + Strength/endurance exercises	12 weeks						
**Falla**	**2006**	Australia	Endurance-strength Group	Sandbag weights	Progressive resistance exercise program for the neck flexors	First stage: 2 weeks; Second stage: 4 weeks	First stage: Weight patients could lift 12 times. From 12 to 15 rep; Second stage: 15 rep x3					
			Cranio-cervical flexion Group	Chattanooga Pressure Biofeedback Unit (Stabilizer)	Cranio-cervical flexion contraction while trying to seep SCM and AS muscle relaxed	6 weeks	hold progressively increasing ranges of cranio-cervical flexion using feedback from an air-filled pressure sensor					
**Falla**	**2008**	Australia	Endurance-strength Group	Sandbag weights	Progressive resistance exercise program for the neck flexors	First stage: 2 weeks; Second stage: 4 weeks	First stage: Weight patients could lift 12 times. From 12 to 15 rep; Second stage: 15 rep x3					
			Cranio-cervical flexion Group	Chattanooga Pressure Biofeedback Unit (Stabilizer)	Cranio-cervical flexion training	6 weeks	hold progressively increasing ranges of cranio-cervical flexion using feedback from an air-filled pressure sensor					
**Falla**	**2013**	Germany	Exercise Group	Chattanooga Pressure Biofeedback Unit (Stabilizer); light weights for the head	A program of exercises commencing with low load motor control exercises and then progressive resistance exercise	Exercises progressed in intensity over the duration of the training; 15 repetitions of a head lift for flexors and extensors; axioscapular control and postural correctio. 10–20 min/day	the number of repetitions was individually tailored to each patient to ensure that they could perform the exercises in a pain-free manner	Control Group		no treatment, patients were not asked to refrain from seeking treatment		
**Mendes-Fernandes**	**2023**	Portugal	Postural global re-education Group		Patients maintain three global postural position for 15–20 min	eight session of 40 min twice a week (4 weeks); Additionally, the patients were asked to perform their exercises at home but without the use of any equipment	Exercises progressed in intensity over the duration of the training					
			Neck specific exercise Group	Laser pointer, Chattanooga Pressure Biofeedback Unit (Stabilizer).	exercises for the cervical and axioscapular muscles and sensoriomotor control exercises	eight session of 40 min twice a week (4 weeks); Additionally, the patients were asked to perform their exercises at home but without the use of any equipment	Exercises progressed in intensity over the duration of the training; 15 repetitions of a head lift for flexors and extensors; axioscapular control and postural correctio. 10–20 min/day					
**Ghaderi**	**2017**	Iran	Exercise Group	Chattanooga Pressure Biofeedback Unit (Stabilizer)	10 session of: Electrotherapy, hot pack, TENS (20 minutes), ultrasound (10 minutes). During the rest of time Craniocervical flexion	10 weeks duration, 3 times per week. Each treatment session took 30-45minutes	From 20mmHg to 30mmHg	Control Group		10 session of: Electrotherapy, hot pack, TENS (20 minutes), ultrasound (10 minutes)		
**Javdaneh**	**2020**	Iran	Scapular exercise Group	Elastic rubber band, dumbbell	Strength exercises	6 weeks, 3 days per week, 40 to 60 minutes per session	Progressive exercises base on sport medicine principles. For the first 2 weeks only unresisted exercises. After 2 weeks dumbbell and elastic rubber bands. Initial load 30% of 1RM increased 10% each week.	Control Group:		A single session where patients were instructed in a home exercise program focused on posture during daily task (pushing, pulling, lifting)		
			Combined (scapular exercise+cognitive functional therapy) Group		Pain science education, tips on sleep hygiene and stress coping strategies; Targeted functional postural and movement training with direction of movement based on movement impairment; Exercises with verbal and mirror feedback	6 weeks	Exercises with feedback moves from verbal to mirror feedback.					
**Jull**	2009	Australia	Strength training group	Weights	Head lift in supine position with weights	6 weeks, twice per day, 10–20 minutes without provoking neck pain	2 stages. First stage (2 weeks): 12–15 repetitions with weight they could lift 12 times. Second stage (4 weeks): 3 set of 10 repetitions, first set at 50% of 10RM, second at 75% of 10RM, third at full 10RM					
			Cranio-cervical flexion training group	Chattanooga Pressure Biofeedback Unit (Stabilizer)	Cranio-cervical flexion training	6 weeks, twice per day, 10–20 minutes without provoking neck pain	From single contraction to progressively increasing mmHg and time					
**Lidegaard**	2013	Denmark	Exercise Group	Thera band	Lateral raise with theraband in the scapular plane	10 weeks, as many repetition as possible for 2 minutes	None	Control Group		They received e-mail once a week on various aspects of general health		
**Lundblad**	1999	Sweden	Physiotherapy Intervention Group		exercises of strength, coordination, endurance, flexibility, and rhythm	50 minutes twice a week in groups of 5 to 8 subjects for 16 weeks. Each subject received also exercises to be practiced at home		Control Group		no treatment		
			Feldenkrais Group:		exercises for increasing awareness about sensory afferents, breaking stereotyped movement patterns and enabling self-care for complaints in neck, shoulders and back	50 minutes per week + home exercises						
**Ma**	2011	China	Biofeedback	Promethus system (portable biofeedback)	An auditory feedback signal warning the subject to try and reduce the UT muscle activity depressing the shoulders or sitting quietly with eyes closed and the shoulder relaxed.	2hours daily while performing computer work for 2 days a week as minimum.	None	Control Group		Standard education booklet about office ergonomics		
			Stretching + neck/shoulder strength training	Thera-band	Strengthening exercises focusing on the neck and shoulder muscles without pain provocation	20 minutes 4 times a day for 6 weeks	None					
			Passive treatment	Endomed model 582 machine	Interferential therapy and hot packs applied to neck and shoulder	Interferential therapy for 20 min, hot packs for 15 minutes twice a week	Intensity was increased to the maximum tolerable without muscle contraction					
**Mehri**	2020	Iran	Experimental Group		progressive resistance exercises for neck muscles, primarily superficial neck flexors and extensor muscles (SCM, AS and CE) to realign the spine, scapula, shoulders, neck and abdomen	12 to 15 repetition of each exercise with 1 minute rest between exercises for 8 weeks	the first 4 weeks 12 times, the last 2 weeks 3 sets of 15	Control group		educational program with correction of posture		
**Yan**	2022	China	Experimental Group	Redcord Suspension System	Cervical flexion and extensor training using a suspension system	Flexion and extension were maintained for 2 minutes. The general duration of the training was around 20–30 min, 3 times per week for 4 weeks.	The Physiotherapist increase the difficulty of the training through shaking the elastic band or through extending the time.	Control group		CGp did not participate in any exercise intervention and maintained a normal daily life.		
**Study**			**Outcome Measures**									
**Author**	**Year**	**Country**	**Pain**		**Disability**	**Neuromuscular adaptation**	**Others**	**Summary results**				
**Beer**	**2012**	Australia	VAS		NDI	EMG (SCM during CCFT)		Pain: No significant difference among groups; Disability: No significant difference among groups; EMG: ExGp. showed less EMG amplitude in SCM at 22 and 26 mmHg during CCFT				
**Borisut**	**2013**	Thailand	VAS		NDI	EMG (UT, CE, SCM, AS ‐ max voluntary contraction)		Pain: Similar significant differences in all groups but CGrp; Disability: Similar significant differences in all groups but CGrp; EMG: Similar reduction of EMG amplitude for all muscles in all groups but CGrp.				
**Falla**	**2006**	Australia	NRS		NDI	Surface EMG from the sternal head of SCM and AS muscles	Strength: MVC isometric cervical flexion for 3 seconds. 10% MVC for 30 seconds, 25% MVC for 20 seconds and 50% MVC for 15seconds.	Pain: Both intervention groups demonstrated a reduction in average intensity of pain; Disability: Both intervention groups demonstrated a reduction in average NDI score; EMG: Endurance-strength training group demonstrated a significant reduction of the MSF initial value and rate of change across all force levels following treatment which was significantly different to the cranio-cervical flexion training group; Strength: Endurance-strength training group demonstrated a greater increase in neck flexion strength compared to the cranio-cervical flexion training group (P<0.05).				
**Falla**	**2008**	Australia	NRS		NDI	Surface EMG from the sternal head of SCM		Pain: Both groups showed a decrease of pain intensity; Disability: Both groups showed a reduction of disability; EMG: no significant change was identified for SCM activity for either group when they performed a repetitive upper limb task				
**Falla**	**2013**	Germany	VAS		NDI	EMG (SCM, Scap ‐ MVCs and SubmaxVC)	Strength: MVCs ‐ neck flexion, extension and lateral flexion	Disability: ExGp. showed a significant reduction; CGp. showed no significant change; EMG: ExGp. Showed less EMG amplitude in SCM and SCap; Strength: Increase of neck flexion strength was statistically significant for the ExGp.				
**Mendes-Fernandes**	**2023**	Portugal	NSR		NDI	EMG (SCM, AS ‐ during the CCFT)		Pain: A significant reduction for both groups; Disability: A significant reduction for both groups; EMG: Postural global re-education group and neck specific exercise group showed a significant reduction in EMG amplitude of SCM and anterior scalene				
**Ghaderi**	**2017**	Iran	VAS		NDI	EMG from SCM, SCap, AS bilaterally and anterior deltoid at the dominant side.		Disability: Both groups showed disability decreased (P<0.001); Pain: Both groups showed neck pain intensity decreased (P<0.001); EMG: activity level of SCM, AS and SCap decreased significantly in stabilization exercises group, whereas it increased significantly in routine exercises group.				
**Javdaneh**	**2020**	Iran	VAS (0-10cm)			EMG of the upper trapezius, lower trapezius, middle trapezius and serratus anterior muscles during shoulder abduction.	Kinesiophobia: Tampa scale	Pain & kinesiophobia: Significant improvements were found for both groups (scapular exercise and multi- disciplinary) in terms of pain intensity and kinesiophobia, but the multidisciplinary group improved significantly more. No significant difference was observed for the control group; EMG: Patients receiving combined scapular exercise and cognitive functional therapy experienced a greater increase in activation of muscles than did those receiving only scapular exercise.				
**Jull**	2009	Australia	NRS		NDI	EMG of DCF, SCM and AS muscles and ROM during the five stages of the CCFT		Pain & Disability: Both exercise groups demonstrated a significant reduction in average pain intensity (NRS) (CCF training, P < 0.001; strength training P < 0.05), and NDI score (C-CF training, P < 0.001; strength training, P < 0.001) but there were no between group differences (both P > 0.05); EMG: Significant changes n EMG was identified only in the CCFT group.				
**Lidegaard**	2013	Denmark	NRS			EMG from the splenius capitis and upper trapezius was recorded during a normal workday.		Pain: Significant improvements were found in the training group; EMG: at 10-week follow-up, training increased average duration of EMG gaps by 71%, EMG gap frequency by 296% and percentage time below 0.5%, and 1.0% EMGmax by 578% and 242%, respectively, during the workday in m. splenius.				
**Lundblad**	1999	Sweden	VAS		Nordic Council of Ministers questionnaire concerning neck and shoulder complaints	EMG (Trapezius part descendent ‐ MVCs shoulder flexion)	Isokinetic endurance muscle test for shoulder flexion	Pain: Feldenkrais and CGp. showed a significant decrease. Physical therapy intervention showed no significant change; Disability: Feldenkrais showed a significant decrease. Physical therapy and CGp. showed no significant change; EMG: Feldenkrais showed greater EMG amplitude in deltoid, trapezius and infraspinatus. CGp. showed greater EMG amplitude in deltoid and infraspinatus. Physical therapy intervention showed no changes; Strength: Shoulder peak torque significantly increased in both ExGp., CGp. showed no significant change.				
**Ma**	2011	China	VAS (10cm)		NDI	EMG during standardized typing task		Disability & Pain: average pain scores and NDI scores of the participants in the biofeedback, active exercise, and passive treatment groups had decreased significantly, and significantly more than in the control group. The average VAS and NDI decreases in the biofeedback group were significantly greater than in the other 3 groups after 6 weeks of intervention. There was no significant difference in the average VAS or NDI results between the active exercise and passive treatment groups. There was also no significant difference in the average VAS or NDI results in the control group comparing preintervention and postintervention; EMG: Reductions in the EMG amplitudes in the CEs and UT muscles were apparent in the biofeedback group postintervention, and these were significantly greater than those observed in the active exercise or passive treatment groups. The control group generally showed no change at all in their EMG amplitudes preintervention and postintervention. A decreasing trend was observed in the right UT muscle, but it was not significant				
**Mehri**	2020	Iran	NPAD		NPAD	EMG (UT, SCM, CE ‐ Root mean square and activation onset)	Motor control test (Patroncini & Luomajoki 2014)	Pain: ExGp. showed a significant reduction; No changes in the CGp; Disability: ExGp. showed a significant reduction; No changes in the CGp; EMG: ExGp. Showed statistically significant less EMG amplitude in UT, SCM and ES on RMS. In the ExGp the activation onset of those muscle is significantly earlier.				
**Yan**	2022	China	VAS		NDI	EMG during CCFT (SCM and UT)		Pain; ExGp. showed a significant reduction; No changes in the CGp; Disability: ExGp. showed a significant reduction; No changes in the CGp; EMG: ExGp. showed a significant reduction in EMG amplitude for both SCM and UT				

%F, female percentage; AS, anterior scalene; CCFT, cranio-cervical flexion test; CE, cervical erector spinae; CGp, control group; DCF, dorsal cervical flexor; EMG, electromyography; ExGp, experimental group; Gp, group; Min, minutes; MVC, maximal voluntary contraction

NDI, neck disability index; NPAD, neck pain and disability scale; NRS, numerical rating scale; RCT, randomised controlled trial; Rep, repetitions; RM, repetition maximum; RMS, root mean square; ROM, range of motion; SE, specific exercises; SCap, splenius capitis; SCM, sternocleidomastoid muscle; SD, standard deviation; TENS, transcutaneus electrical nerve stimulation, UT, upper trapezius; VAS, visual analog scale; VC, voluntary contraction

**Table 2:** Exercise characteristics.

Exercise Training	Study	What	Duration	Frequency		Intensity				
				Supervised (per week)	Home exercise program	Reps	Sets	Load	Rest	Tempo
**Functional posture exercise**	Beer 2012	Upright posture in a neutral lumbo-pelvic position and then gently lengthen the cervical spine by imagining they are lifting the base of their skull from the top of their neck	2 weeks	3 in two weeks	Everyday every 15-20min	1	1	-	Not reported	hold 10sec
**Strength/endurance exercises**	Borisut 2013	Progressive resistance exercise program for the neck muscles, especially targeting the superficial neck flexor and extensor muscles (SCM, AS and CE). Neck flexion and extension were performed in the supine and prone positions, respectively, with the head supported in a comfortable resting position. Subjects slowly moved the head and neck through the total range of motion avoiding discomfort or symptom reproduction.	12 weeks (2 phases: 4 and 8 weeks)	not reported	1 per day	Phase1: 12 | Phase2: 15	Phase1: 1 | Phase2: 3	A weight that patients could lift 12 times	1min	-
**CCFT**	Borisut 2013	CCFT performed in supine from 20mmHg to 22-30mmHg	12 weeks	not reported	1 per day	15	-	not reported	10sec	hold 10sec
**Endurance strength training**	Falla 2006, Falla 2008, Jull 2009	Endurance strength training of the cervical flexor muscles. Progressive resistance exercise performed in supine position	Phase 1: 2 weeks | Phase 2: 4 weeks	Once per week	Twice per day	Phase 1: 12–15	Phase 1: 1	A weight that patients could lift 12 times	1min	-
						Phase 2: 15	Phase 2: 3			
**CCFT**	Falla 2006, Falla 2008, Jull 2009	Low load training of the cranio-cervical flexor muscles with stabilizer biofeedback	6 weeks	Once per week	Twice per day	10	Five increment stages	From 22 to 30 mmHg	30sec between sets, 3-5sec between reps	hold 10sec
**Program of neck specific exercises**	Falla 2013,	Exercises for the cervical and axioscapular region and sensoriomotor control exercises with visual feedback with a laser pointer. Specific low-load exercises for both the deep neck flexors, performed in supine lying, using the Stabilizer and deep neck extensors as participants performed isolated neck extension in a prone position. This was then progressed to higher load exercise.	Total 8 weeks	2 times per week	2 per day	up to 15, patient tailored	patient tailored	Level and amount of set/reps were tailored to each patient	no rest	Phase 2: 3sec
	Mendes Fernandes 2023	Phase 1: CCFT in supine position with stabilizer | Phase 2: head weight exercises in flexion and extension	Phase 1: 6 weeks | Phase 2: 2 weeks							
**Global Postural Re-education**	Mendes Fernandes 2023	Three position described by Souchard: 1) supine with shoulder abducted to 30° and forearms supinated, the patient extend hips and knees 2) supine with hips at 90° with gradual knee extension 3) Upright posture in standing	8 weeks	2 times per week	Everyday	not reported	15–20 min exercise 1 & 2, 5 min exercise 3	Exercises progressed in intensity over the duration of the training	not reported	5–10 sec of isometric contraction of the antagonist muscles
**Stabilization neck exercise group**	Ghaderi 2017	Stabilization exercises targeted the deep flexor muscles of neck rather than the superficial flexor muscles emphasizing Craniocervical Flexion with stabilizer biofeedback + electrotherapy for 10 sessions including Hot Pack (HP)/Transcutaneous Electrical Nerve Stimulation (TENS) for 20 min and ultrasound (US) for paraspinal muscles for 10 min bilaterally	10 weeks	3 times per week	-	not reported	not reported	From 22 to 30 mmHg	not reported	not reported
**Routine exercise group**	Ghaderi 2017	progressive resistive exercises + electrotherapy for 10 sessions including Hot Pack (HP)/Transcutaneous Electrical Nerve Stimulation (TENS) for 20 min and ultrasound (US) for paraspinal muscles for 10 min bilaterally	10 weeks	3 times per week	-	not reported	not reported	30% of MVC	not reported	not reported
**Scapular exercise group**	Javdaneh 2020	Scapulothoracic exercises included specific exercises for the muscles affecting scapular orientation related to neck pain. Non-resistive scapular upward rotation exercise done with an elastic rubber band and the dumbbell exercises included wall facing arm lift, backward rocking arm lift, arm raise overhead in line with the lower trapezius muscle fibres, shoulder abduction in the plane of the scapula above 120°, shoulder shrug. Also, levator scapulae and pectoralis minor muscle stretching was performed. 10 minutes warm-up exercises, 30 minutes exercises of scapular, 5 minutes cool-down. Stretching exercises were performed once a day (three days per week, for six weeks) and three sets of 10 to 30 seconds.	6 weeks (First 2 weeks unresisted exercises; Then 4 weeks with dumbbells and elastic band)	Exercise: 3 days per week	-	10 to 15	3	Exercise with dumbbell progressive load 30% 1RM + 10% each week	30 sec	not reported
**Cognitive functional therapy and scapular exercise group (multidisciplinary group)**	Javdaneh 2020	Cognitive component: patient education based on ongoing pain science Functional movement exercises: provide patients with alternative strategies to normalize their postural and movement behaviors. Scapular exercises with verbal and mirror feedback	6 weeks (First 2 weeks unresisted exercises Then 4 weeks with dumbbells and elastic band)	Exercise: 3 days per week	-	10 to 15	3	Exercise with dumbbell progressive load 30% 1RM + 10% each week	30 sec	not reported
**Lateral raise exercise**	Lidegaard 2013	Shoulder abduction in the scapular plane with elastic tubing	10 weeks	-	5 times per week	AMRAP (Exercise was performed to failure)	1	3 elastic bands: red (22N), green (29N), blue (40N). During the initial 2 weeks, they used moderate resistance (red women and green men). After 2 weeks progressed to a higher level of resistance, receiving instructions to increase resistance when they could perform more than a specified number of repetitions according to the following scheme; 22, 20, 18, and 16 repetitions, respectively, at the 2nd (eg, green for women), 3rd, 4th, and 5th (eg, red + blue for women) levels of resistance.	-	2 seconds
**Physiotherapy intervention**	Lundblad 1999	Stabilizing exercises and isolated and relaxed shoulder movements	16 weeks	twice a week per 50min	not reported	not reported	not reported	not reported	not reported	not reported
**Feldenkrais intervention**	Lundblad 1999	Increasing awareness about sensory afferens & breaking stereotyped movement patterns	16 weeks	12 times per 50 min	4 times per 50min	not reported	not reported	not reported	not reported	not reported
**Stretching + neck/shoulder strength training**	Ma 2011	Standardized exercise program including both stretching exercises and strengthening exercises using a Thera-band, focusing on the neck and shoulder muscles	6 weeks	first session	no longer than 20 minutes 4 times a day	not reported	not reported	not reported	not reported	not reported
**Corrective exercise program**	Mehri 2020	Progressive resistance exercise program for the neck muscles, primarily the superficial neck flexor and extensor muscles (SCM, AS, and CE)	Phase 1: 4 weeks | Phase 2: 4 weeks	3 days per week	-	Phase1: 12–15 | Phase2: 15	Phase1: 1 | Phase2: 3	progressive resistance exercise program: three phases during the exercise 1. slow control; 2. muscle endurance; 3. muscle strength	1min	30-60sec
**Redcord Suspension System**	Yan 2022	Cervical flexion and extensor training using a suspension system	4 weeks	3 days per week	20–30 min	2min	not reported	not reported	not reported	not reported

1RM, 1 repetition maximum; AMRAP, as many repetition as possible; AS, anterior scalene; CCFT, cranio-cervical flexion test; CE, cervical erector spinae; SCM, sternocleidomastoid muscle

Mean and standard deviation data was extracted for each outcome measure (Table 3). If the mean and standard deviation were not available but this data was presented in figures, we used the WebPlotDigitizer [25] to estimate these data.

**Table 3:** Outcome measures and their change following the interventions.

**EMG–AMPLITUDE**																						
**Author**	**Treatment**	**Outcome measure**	**Mean Pre**		**SD Pre**		**Mean Post**		**SD Post**		**Pre-post Estimate effect [95% CIs]**				**Mean post**		**SD post**		**Treatment-control Estimate effect [95% CIs]**			
**Beer 2012**	Postural exercise	SCM during CCFT:	6.5		5.3		2.8		3.5		0.043		*		8.3		10.9		0.15			
		22 mmHg	16.0		12.3		7.5		6.3		0.053		*		19.4		16.1		0.04		*	
		24 mmHg	28.6		9.5		11.1		7.9		0.003		*		27.7		22.3		0.04		*	
		26 mmHg	32.4		12.6		21.5		14.8		0.06				47.3		31		0.03		*	
		30 mmHg	45.1		29.8		30.6		21.2		Not statistically significant				59.2		36.1		0.04		*	
**Borisut 2013**	Strength-endurance	During typing task									P<0.05		*						P<0.05		*	
		UT	22.53		13.33		5.90		3.09						21.98		13.68					
		CE	14.73		6.51		5.91		1.26						13.50		4.18					
		SCM	7.75		4.24		2.19		0.93						8.59		8.15					
		AS	12.38		6.62		3.38		0.81						12.26		4.15					
**Borisut 2013**	CCF training	During typing task									P<0.05		*						P<0.05		*	
		UT	19.45		10.42		6.3		3.34						21.98		13.68					
		CE	17.74		7.76		5.88		0.94						13.50		4.18					
		SCM	7.24		8.06		1.42		0.39						8.59		8.15					
		AS	13.41		8.97		3.61		1.33						12.26		4.15					
**Borisut 2013**	Strength+CCF training	During typing task									P<0.05		*						P<0.05		*	
		UT	15.43		9.12		4.6		1.76						21.98		13.68					
		CE	14.46		6.82		6.57		1.24						13.50		4.18					
		SCM	7.13		3.59		2.37		1.03						8.59		8.15					
		AS	10.60		4.91		3.44		0.57						12.26		4.15					
**Falla 2006**	Endurance-strength						Pre-to post		Pre-to post		P<0.05		*		No control group		No control group		No control group		-	
		SCM @ 50% MVC	129		10.3		-7.8		7.5													
		SCM @ 25% MVC	125		9.05		-17.1		10.8													
		SCM @ 10% MVC	116		14.9		-11.8		10.7													
		AS @ 50% MVC	131		15.6		-13.4		11.4													
		AS @ 25% MVC	124		17.8		-15.0		14.9													
		AS @ 10% MVC	113		18.7		-12.7		12.7													
**Falla 2006**	CCF training						Pre-to post		Pre-to post		P>0.05				No control group		No control group		No control group		-	
		SCM @ 50% MVC	126		16.9		-4.3		7.1													
		SCM @ 25% MVC	121		17.5		-1.0		11.6													
		SCM @ 10% MVC	116		19.3		-0.8		12.1													
		AS @ 50% MVC	129		20.5		-3.0		7.2													
		AS @ 25% MVC	125		18.6		-5.7		11.9													
		AS @ 10% MVC	115		20.2		-3.0		10.8													
**Falla 2008**	Endurance-strength	@upper arm task	Pre		Pre		Pre- to post		Pre- to post		P>0.05				No control group		No control group		No control group		-	
		SCM root mean square	Left	Right	Left Right		Left	Rigth	Left	Rigth												
		10s	11.7	8.8	8.7 5.9		-1.1	1.8	6.9	8.5												
		60s	12.6	10.0	9 8.4		0.6	-0.0	9.3	9.2												
		120s	12.7	11.0	9.7. 11.1		-0.9	-1.1	9.0	10.9												
		10s post task	12.8	8.2	10.9 5.7		-2.1	-0.9	10.3	6.8												
**Falla 2008**	CCF training	@upper arm task	Pre		Pre		Pre- to post		Pre- to post		P>0.05				No control group		No control group		No control group		-	
		SCM root mean square	Left	Right	Left	Rigth	Left	Rigth	Left	Rigth												
		10s	13.3	13.4	10.0	9.0	-0.0	-1.6	10.4	7.9												
		60s	15.2	14.4	10.5	9.4	-1.3	-1.1	8.6	7.4												
		120s	14.3	15.5	8.4	10.3	1.4	-1.1	8.9	8.1												
		10s post task	12.8	10.0	9.3	7.0	0.8	0.0	9.4	9												
**Falla 2013**	Neck specific therapeutic exercises	@MVCs SCM and SCap (average)	27.4		18.0		18.2		10.2		P<0.05		*		26.3		17.3		not reported			
**Fernandes 2023**	Global postura Re-education	RMS during CCFT	SCM	As	SCM	As	SCM	As	SCM	As	P<0.05		*		No control group		No control group		No control group		-	
		22mmHg	27.7	22.2	5.3	3.6	19.9	17.0	3.3	2.4												
		24mmHg	29.1	25.2	3.5	2.8	20.9	18.6	2.5	2.2												
		26mmHg	31.5	27.1	4.4	4.1	23.0	23.3	2.7	3.3												
		28mmHg	33.6	29.4	3.4	4.1	25.6	25.0	3.0	3.4												
		30mmHg	38.3	32.7	4.8	4.0	28.6	26.3	3.8	3.4												
**Fernandes 2023**	Neck specific therapeutic exercises	RMS during CCFT	SCM	As	SCM	As	SCM	As	SCM	As	P<0.05		*		No control group		No control group		No control group		-	
		22mmHg	36.1	34.6	3.8	4.2	26.1	26.3	2.8	2.8												
		24mmHg	38.8	35.6	3.2	3.9	30.1	27.2	3.5	2.8												
		26mmHg	45.4	42.7	4.5	4.9	35.1	34.5	3.7	3.5												
		28mmHg	48.8	46.7	4.9	4.8	37.5	35.2	3.6	3.8												
		30mmHg	53.3	52.3	4.6	6.6	41.3	40.4	4.2	5.4												
**Ghaderi 2017**	CCF training	RMS during CCFT @30mmHg	Left	Rigth	Left	Rigth	Left	Rigth	Left	Rigth	P < 0,05		*		Left	Rigth	Left	Rigth	P < 0,05		*	
		SCM	75.9	69.8	5.7	6	23.2	32.9	4.6	4					67.3	64.5	5.6	4.8				
		AS	84.3	86.6	11.1	7.7	32.9	40.6	4.6	6.9					91.2	92.1	5.6	5.9				
		SCap	82.9	90.6	6.7	8.2	30.6	40.9	4.8	4.9					95.2	88.7	6.0	5.9				
**Javdaneh 2020**	Scapular stabilizer exercises	% peak dynamic activity	60°	120°	60°	120°	60°	120°	60°	120°	60°	120°	60°	120°	60°	120°	60°	120°				
		UT	35.75	42.50	15.05	21.3	50.08	57.16	22.78	26.2	0.001	0.001	*	*	34.58	40.83	17.62	15.66	0.003	0.009	*	*
		LT	22.82	28.53	7.20	19.2	26.10	51.16	11.99	18.9	0.43	0.001		*	21.96	28.53	7.56	14.2	0.009	0.040	*	*
		MT	18.22	29.58	4.43	16.8	22.40	47.28	4.19	19.87	0.21	0.004		*	18.22	28.56	4.58	13.8	0.75	0.027		*
		SA	15.64	28.53	12.3	19.20	25.49	50.93	13.4	18.61	0.047	0.045	*	*	16.10	28.53	12.45	19.20	0.025	0.038	*	*
**Javdaneh 2020**	Scapular stabilizer exercises + cognitive functional therapy	% peak dynamic activity	60°	120°	60°	120°	60°	120°	60°	120°	60°	120°	60°	120°	60°	120°	60°	120°				
		UT	37.93	41.75	19.14	18.78	54.73	73.41	21.97	29.55	0.001	0.001	*	*	34.58	40.83	17.62	15.66	0.001	0.001	*	*
		LT	22.00	27.50	7.66	18.61	34.92	55.33	11.57	19.24	0.009	0.001	*	*	21.96	28.53	7.56	14.2	0.005	0.030	*	*
		MT	18.83	28.50	5.95	15.2	26.05	60.25	7.2	25.6	0.045	0.001	*	*	18.22	28.56	4.58	13.8	0.38	0.004		*
		SA	16.08	27.50	12.36	18.61	35.41	55.33	13.08	19.24	0.001	0.001	*	*	16.10	28.53	12.4	19.20	0.001	0.030	*	*
**Jull 2009**	CCF training	During CCFT 30 mmHg	Left	Rigth	Left	Rigth	Left	Rigth	Left	Rigth	P<0.001		*		No control group		No control group		No control group			
		SCM	73.2	68.1	26.4	21.1	29.8	29.9	27.5	20.0												
		AS	82.3	83,4	40.1	39.6	27.5	36.6	17.4	22.8												
		DCFs	41.0		19.3		59.3		18.8													
**Jull 2009**	Strength training	During CCFT 30 mmHg	Left	Rigth	Left	Rigth	Left	Rigth	Left	Rigth	P>0.05				No control group		No control group		No control group		-	
		SCM	65.0	59.4	26.5	21.1	57.1	56.1	26.1	27.7												
		AS	76.0	84.7	33.8	29.8	64.9	73.6	26.6	33.0												
		DCFs	40.5		22.5		49.8		22.9													
**Lidegaard 2013**	Strength training: shoulder lateral raise		Before training	After trianing	Before training	After trianing	Before training	After trianing	Before training	After trianing	Before training	After trianing	Before training	After trianing	Before training	After trianing	Before training	After trianing	Before training	After trianing	Before training	After trianing
		Trapezius during maximal voluntary arm contraction	8.5	7.4	4.4–11,6	1.4–10.9	8.2	7.6	6.3–15.3	4.8–15.2	P>0.05	P<0.05		*	3.7	1.1	1.1–13.2	0.5–5.8	P>0.05	P<0.05		*
		splenius capitis during maximal voluntary arm contraction	3.1	5.0	5.4–15.2	2.7–7.8	12.3	8.0	4.4–15.2	3,5–14.5	P>0.05	P>0.05			2.2	1.3	1.2–6.4	0.5–6.5	P>0.05	P>0.05		
**Lundblad 1999**	Mixed physiotherapy exercises	@MVCs shoulder flexion									Not statistically significant								Not statistically significant			
		Trapezius	67.7		9.6		66.8		8.6						74.1		11.1					
		Deltoid	62.7		11.0		65.4		12.8						66.7		8.7					
		Infraspinatus	67.1		15.1		76.4		14						82.5		10.3					
		Biceps brachii	62.8		7.7		63.9		13.4						65.7		11.4					
**Lundblad 1999**	Feldenkrais	@MVCs shoulder flexion																	Not statistically significant			
		Trapezius	65.7		8.2		70.6		7.3		Statistically significant		*		74.1		11.1					
		Deltoid	61.9		6.5		65		8.7		Statistically significant		*		66.7		8.7					
		Infraspinatus	70.8		10.1		85.4		17.7		Statistically significant		*		82.5		10.3					
		Biceps brachii	64.5		14.4		64.2		11.7		Not statistically significant				65.7		11.4					
**Ma 2011**	Stretching + neck/shoulder strength training	During tapping task	Left	Rigth	Left	Rigth	Left	Rigth	Left	Rigth	Not statistically significant				Left	Rigth	Left	Rigth	Not statistically significant			
		CEs @ 90uV	13.2	15.9	7.0	8.7	16.1	15.3	8.2	7.6					16.8	13.6	9.1	8.6				
		UT @ 90uV	32.8	36.0	19,2	29.5	31.3	23.2	38,7	38.6					20.0	20.6	10.7	15				
**Mehri 2020**	Resistance training	@RMS											*									
		UT	48.32		8.41		39.2		6.26		P = 0.005				48.26		7.45		0.037		*	
		SCM	41.4		6.72		35.3		4.12		P = 0.024				44.3		8.24		0.024		*	
		ES	55.22		9.21		43.1		11.23		P = 0.019				56.1		8.52		0.019		*	
		@activation onset																				
		UT	165.42		15.78		147.65		15.75		P = 0.008				158.31		17.51		0.008		*	
		SCM	142.38		18.77		118.34		16.55		P = 0.005				127.82		20.45		0.005		*	
		ES	156.38		16.14		142.85		19.73		P = 0.001				151.31		12.35		0.001		*	
**Yan 2022**	Redcord Suspension System	During CCFT	SCM	UT	SCM	UT	SCM	UT	SCM	UT	Not reported				SCM	UT	SCM	UT				
		22mmHg	16.36	38.02	3.56	2.98	8.41	16.59	2.27	4.03					15.74	36.21	3.11 / 2.45	2.45	P<0.001	P<0.001	*	*
		24mmHg	20.84	42.87	3.22	3.27	?	23.43	?	4.67					?	40.17	? / 1.73	1.73	?	P<0.001	?	*
		26mmHg	26.38	47.03	4.04	2.74	15.78	27.82	3.33	3.41					29.50	43.28	6.53 / 3.34	3.34	P<0.001	P<0.001	*	*
		28mmHg	32.08	49.73	4.20	2.72	19.25	30.88	3.57	3.20					33.07	45.22	7.70 / 4.89	4.89	P<0.001	P<0.001	*	*
		30mmHg	36.29	53.7	5.47	3.21	22.16	35.02	5.52	3.50					37.56	50.28	7.53 / 4.27	4.27	P<0.001	P<0.001	*	*
**PAIN**																						
**Author**	**Treatment**	**Outcome measure**	**Mean Pre**		**SD Pre**		**Mean Post**		**SD Post**		**Pre-post Estimate effect [95% CIs]**				**Mean post**		**SD post**		**Treatment-control Estimate effect [95% CIs]**			
**Beer 2012**	Postural exercise	VAS	3		1.7		3		2.8		P > 0.05				2.9		1.2		0.92			
**Borisut 2013**	Strength-endurance	VAS (0–100)	55		10.98		38.68		9.49		P<0.002		*		61.32		11.29		not reported			
**Borisut 2013**	CCF training	VAS (0–100)	56.04		22.66		43.04		18.56		P<0.002		*		61.32		11.29		not reported			
**Borisut 2013**	Strength+CCF training	VAS (0–100)	61.48		16.68		16.88		7.75		P<0.002		*		61.32		11.29		not reported			
**Falla 2006**	Endurance-strength	NRS (0–10) (post-pre)	4.7		2.0		-1.1		2.8		Not reported				No control group		No control group		No control group		-	
**Falla 2006**	CCF training	NRS (0–10) (post-pre)	3.6		2.0		-0.9		2.3		Not reported				No control group		No control group		No control group		-	
**Falla 2008**	Endurance-strength	NRS (0–10) (post-pre)	4.7		2.0		-1.1		2.8		P>0.05				No control group		No control group		No control group		-	
**Falla 2008**	CCF training	NRS (0–10) (post-pre)	3.05		2.00		-0.9		2.4		P>0.05				No control group		No control group		No control group		-	
**Falla 2013**	Neck specific therapeutic exercises	VAS (0–10) (post-pre)	18.2		7.4		-1.7		2.2		Not reported				-0.3		2.1		P<0.05		*	
**Fernandes 2023**	Global postura Re-education	NPRS	6.16		1.40		2.56		1.36		P<00.5		*		No control group		No control group		No control group		-	
**Fernandes 2023**	Neck specific therapeutic exercises	NPRS	6.04		1.65		2.24		1.23		P<00.5		*		No control group		No control group		No control group		-	
**Ghaderi 2017**	CCF training	VAS (0–100)	61.35		27.95		21.73		15.9		P<0.001		*		20.73		11.3		0.444			
**Javdaneh 2020**	Scapular stabilizer exercises	VAS (0–100)	56.75		8.54		28.41		8.78		P<0.001		*		57.41		8.95		0.003		*	
**Javdaneh 2020**	Scapular stabilizer exercises + cognitive functional therapy	VAS (0–100)	58		7.95		8.05		6.09		P<0.001		*		57.41		8.95		0.001		*	
**Jull 2009**	CCF training	NRS (0–10) (post-pre)	4.5		1.6		-1.7		2.0		P<0.001		*		No control group		No control group		No control group		-	
**Jull 2009**	Strength training	NRS (0–10) (post-pre)	4.2		2.1		-1.0		3.3		P<0.001		*		No control group		No control group		No control group		-	
**Lidegaard 2013**	Strength training: shoulder lateral raise	NRS	3.44		1.40		2.04		1.60		P<0.01		*		3.45		1.99		P<0.01		*	
**Lundblad 1999**	Physiotherapy	VAS	1.2		1		0.9		1.3		Not statistically significant				1.1		1.4		Not statistically significant			
**Lundblad 1999**	Feldenkrais	VAS	1.5		1		0.3		0.6		P<0.05		*		1.1		1.4		Not statistically significant			
**Ma 2011**	Strength training	VAS	4,75		1.59		2.10		1.34		P<0.05		*		4.75		1.53		P<0.05		*	
**Mehri 2020**	Resistance training	NPAD	29.64		2.93		24.23		5.57		0.002		*		27.07		4.25		P<0.05		*	
**Yan 2022**	Redcord Suspension System	VAS (0–10)	5.67		1.37		3.83		1.27		P<0.001		*		5.46		1.33		0.005		*	
**DISABILITY**																						
**Author**	**Treatment**	**Outcome measure**	**Mean Pre**		**SD Pre**		**Mean Post**		**SD Post**		**Pre-post Estimate effect [95% CIs]**				**Mean post**		**SD post**		**Treatment-control Estimate effect [95% CIs]**			
**Beer 2012**	Postural exercise	NDI	18.1		9.0		17.8		11.9		Not statistically significant				21.8		11.9		0.46			
**Borisut 2013**	Strength-endurance	NDI	28.2		5.56		14.69		4.64		P = 0.001		*		31.56		5.14		Statistically significant		*	
**Borisut 2013**	CCF training	NDI	29.96		4.51		14.41		4.94		P = 0.001		*		31.56		5.14		Statistically significant		*	
**Borisut 2013**	Strength+CCF training	NDI	29.23		5.27		15.71		3.01		P = 0.001		*		31.56		5.14		Statistically significant		*	
**Falla 2006**	Endurance-strength	NDI (post-pre)	9.8		3.3		-2.8		4.0		Not reported				No control group		No control group		No control group		-	
**Falla 2006**	CCF training	NDI (post-pre)	10.4		3.4		-3.5		4.8		Not reported				No control group		No control group		No control group		-	
**Falla 2008**	Endurance-strength	NDI (post-pre)	10.1		3.0		-2.8		4.0		P>0.05				No control group		No control group		No control group		-	
**Falla 2008**	CCF training	NDI (post-pre)	9.8		3.3		-3.7		4.7		P>0.05				No control group		No control group		No control group		-	
**Falla 2013**	Neck specific therapeutic exercises	NDI (post-pre)	18.2		7.4		14.1		6.5		P<0.01		*		16.6		7.4		P<0.05		*	
**Mendes Fernandes 2023**	Global postura Re-education	NDI	15.52		5.42		11.24		5.58		P<0.01		*		No control group		No control group		No control group		-	
**Mendes Fernandes 2023**	Neck specific therapeutic exercises	NDI	16.08		5.34		11.80		6.09		P<0.01		*		No control group		No control group		No control group		-	
**Ghaderi 2017**	CCF training	NDI	31.25		12.11		15.25		9.03		P<0.001		*		18.54		12.5		0.306			
**Jull 2009**	CCF training	NDI (post-pre)	11		2.7		-5.0		4.2		P<0.001		*		No control group		No control group		No control group		-	
**Jull 2009**	Strength training	NDI (post-pre)	9.6		3.1		-3.5		2.3		P<0.001		*		No control group		No control group		No control group		-	
**Lundblad 1999**	Physiotherapy	Nordic Council of Ministers questionnaire	1.3		1.0		1.3		1.1		Not statistically significant				1.2		1		Not statistically significant			
**Lundblad 1999**	Feldenkrais	Nordic Council of Ministers questionnaire	1.2		0.9		1.0		1.0		Not statistically significant				1.2		1		Not statistically significant			
**Ma 2011**	Strength training	NDI	16.5		5.13		10.33		2.23		P<0.05		*		14.82		2.87		P<0.05		*	
**Mehri 2020**	Resistance training	NPAD	29.64		2.93		24.23		5.57		0.002		*		27.07		4.25		P<0.05		*	
**Yan 2022**	Redcord Suspension System	NDI	27.50		10.68		19.17		9.15		P<0.001		*		28.62		10.02		0.022		*	
**STRENGTH**																						
**Author**	**Treatment**	**Outcome measure**	**Mean Pre**		**SD Pre**		**Mean Post**		**SD Post**		**Pre-post Estimate effect [95% CIs]**				**Mean post**		**SD post**		**Treatment-control Estimate effect [95% CIs]**			
**Falla 2013**	Neck specific therapeutic exercises described by Jull (2008)	@MVCs																				
		flexion	94.7		24.3		104.6		34.2				*		88.6		34.4					
		extension	176.2		47.8		174.9		50						162.9		57.2					
		right lateral flexion	122.3		42.7		122.8		43.7						108.5		39.8					
		left lateral flexion	122.95		32.6		126.2		38.7						111.5		41					
**Lundblad 1999**	Physiotherapy	Isokinetic endurance muscle test for shoulder flexion	34		7.9		37		5.9		Not statistically significant											
**Lundblad 1999**	Feldenkrais	Isokinetic endurance muscle test for shoulder flexion	33.6		5.4		37		5.9		Not statistically significant											
**ENDURANCE**																						
**Author**	**Treatment**	**Outcome measure**	**Mean Pre**		**SD Pre**		**Mean Post**		**SD Post**		**Pre-post Estimate effect [95% CIs]**				**Mean post**		**SD post**		**Treatment-control**			
																			**Estimate effect [95% CIs]**			
**Ghaderi 2017**	CCF training	CCFT (seconds)	19.53		14.7		73.59		17.7		<0.001		*		41.23		26.9		P = 0.365			
**EMG ‐ TIMING MUSCLE ACTIVITY (LANTENCY in milliseconds)**																						
**Author**	**Treatment**	**Outcome measure**	**Mean Pre**		**SD Pre**		**Mean post or Pre-post**		**SD Pre-to post**		**Pre-post Estimate effect [95% CIs]**				**Mean post**		**SD post**		**Treatment-control**		**Treatment-control Estimate effect [95% CIs]**	
**Jull 2009**	CCF training	Arm flexion	-		-		Left	Rigth	Pre-post		Not statistically significant				No control group		No control group		-		-	
		DCF					-9.10		6,47													
		SCM					-7.97	-9.05	6.68	6.78												
		As					-15.13	-7.06	9.16	6.73												
		Arm extension					Left	Rigth	Pre-post													
		DCF					-12.93		4.95													
		SCM					-8.62	-8.78	4.31	3.45												
		As					-12.33	-12.87	3.07	5.5												
**Jull 2009**	Strength training	Arm flexion	-		-		Left	Rigth	Left	Rigth	Not statistically significant				No control group		No control group		-		-	
		DCF					-3.18		7.81													
		SCM (L/R)					-7.97	-10.88	6.52	6.03												
		AS (L/R)					-8.13	-7.16	7.06	6.20												
		Arm extension					Left	Rigth	Left	Rigth												
		DCF					-3.07		7.70													
		SCM					-7.32	-8.40	6.74	6.57												
		As					-14.27	-11.63	6.9	7.11												
**Mehri 2020**	Resistance training	UT	165.42		15.78		147.65		15.75		P = 0.004		*		158.31		17.51		P = 0.009		*	
		SCM	142.38		18.77		118.34		16.55		P = 0.003		*		127.82		20.45		P = 0.005		*	
		ES	156.38		16.14		142.85		19.73		P = 0.004		*		151.31		12.35		P = 0.001		*	
**Ghaderi 2017**	CCF training		Left	Rigth	Left	Rigth	Left	Rigth	Left	Rigth	Not reported				Left	Rigth	Left	Rigth		
		SCM	83.86	91.54	9.25s	8.26	76.57	84.25	8.27	9.45					66.14	68.50	6.69	7.48	P<0.005	*
		AS	96.46	89.57	10.43	9.25	78.45	84.25	12.20	9.45					69.29	89.96	8.27	11.81	P<0.005	*
		Scap	69.69	63.39	11.41	9.44	61.61	59.06	11.42	12.59					80.31	62.20	12.41	5.12	P<0.005	*
**EMG ‐ MUSCLE FATIGABILITY**																						
**Author**	**Treatment**	**Outcome measure**	**Mean Pre**		**SD Pre**		**Mean Pre-to post**		**SD Pre-to post**		**Pre-post Estimate effect [95% CIs]**				**Mean post**		**SD post**		**Treatment-control**		**Treatment-control Estimate effect [95% CIs]**	
**Falla 2006**	Endurance-strength	SCM @ 50% MVC	90.1		34.4		0.40		0.30		P<0.05		*		No control group		No control group		No control group		-	
		SCM @ 25% MVC	61.9		29.7		0.45		0.39													
		SCM @ 10% MVC	41.3		30.5		0.26		0.28													
		AS @ 50% MVC	84.8		36.1		0.26		0.29													
		AS @ 25% MVC	56.1		29.7		0.10		0.26													
		AS @ 10% MVC	38.1		27.5		0.07		0.23													
**Falla 2006**	CCF training	SCM @ 50% MVC	80.2		30.7		0.06		0.43		P>0.05				No control group		No control group		No control group		-	
		SCM @ 25% MVC	51.0		19.9		0.07		0.27													
		SCM @ 10% MVC	32.9		16.9		0.01		0.24													
		AS @ 50% MVC	41.3		31.9		-0.01		0.24													
		AS @ 25% MVC	84.8		22.4		-0.04		0.20													
		AS @ 10% MVC	32.3		17.1		-0.08		0.22													

AS, anterior scalene; CCFT, cranio-cervical flexion test; CE, cervical erector spinae; CI, confindence interval; DCF, dorsal cervical flexor; EMG, electromyography; ES, erector spinae; LT, lower trapezius; MVC, maximal voluntary contraction; MT, middle trapezius; NDI, neck disability index; NPAD, neck pain and disability scale; NRS, numerical rating scale; RMS, root mean square; SA, serratus anterior; SCap, splenius capitis; SD, standard deviation; SCM, sternocleidomastoid muscle; UT, upper trapezius; VAS, visual analog scale

### Risk of bias assessment

To assess the strength and quality of evidence, two reviewers (AMD and DA) assessed the risk of bias of each study using the Cochrane Collaboration’s risk of bias assessment tool [26]. The Cochrane risk of bias tool assesses bias across 5 domains: Bias arising from the randomization process, bias due to deviations from intended interventions, Bias due to missing outcome data, Bias in measurement of the outcome, bias in selectin of the reported result. The 5 domains are rated as “high,” “low,” or “unclear” risk. Lastly an overall risk of bias has been reported.

### Data synthesis

A narrative synthesis was firstly conducted, describing the type of study, variations within interventions, study design, outcome measures, comparability, and comments about the study’s overall quality. Data are reported as “mean ± standard deviation”; p-values have been reported when provided by authors.

A meta-analysis based on EMG data was conducted including 6 of the 14 studies for a total of 9 interventions. This could be achieved for the sternocleidomastoid (SCM) EMG data only. The I-square, heterogeneity measure score, was 0.96 and the CI was -1.715, therefore a meta-analysis could be conducted.

### Certainty of evidence

The Grading of Recommendations Assessment, Development and Evaluation (GRADE) [27] method was used to appraise the certainty of the pooled evidence. The overall certainty of evidence for each type of exercise and neuromuscular adaptation was rated using GRADE. The quality of evidence was assessed as “high”, “moderate”, “low” or “very low” by two independent reviewers (AMD/DA).

We rated every article included in this systematic review considering the following criteria described by Guyatt et al. [28]: “risk of bias”, “inconsistency”, “indirectness”, “imprecision” and “publication bias”. Moreover, as suggested by Guyatt et al. [29] we downrated the quality of evidence of methods when the total sample size was low (<24 participants) as this might affect “imprecision”.

## Results

### Study selection

**Fig 1:**
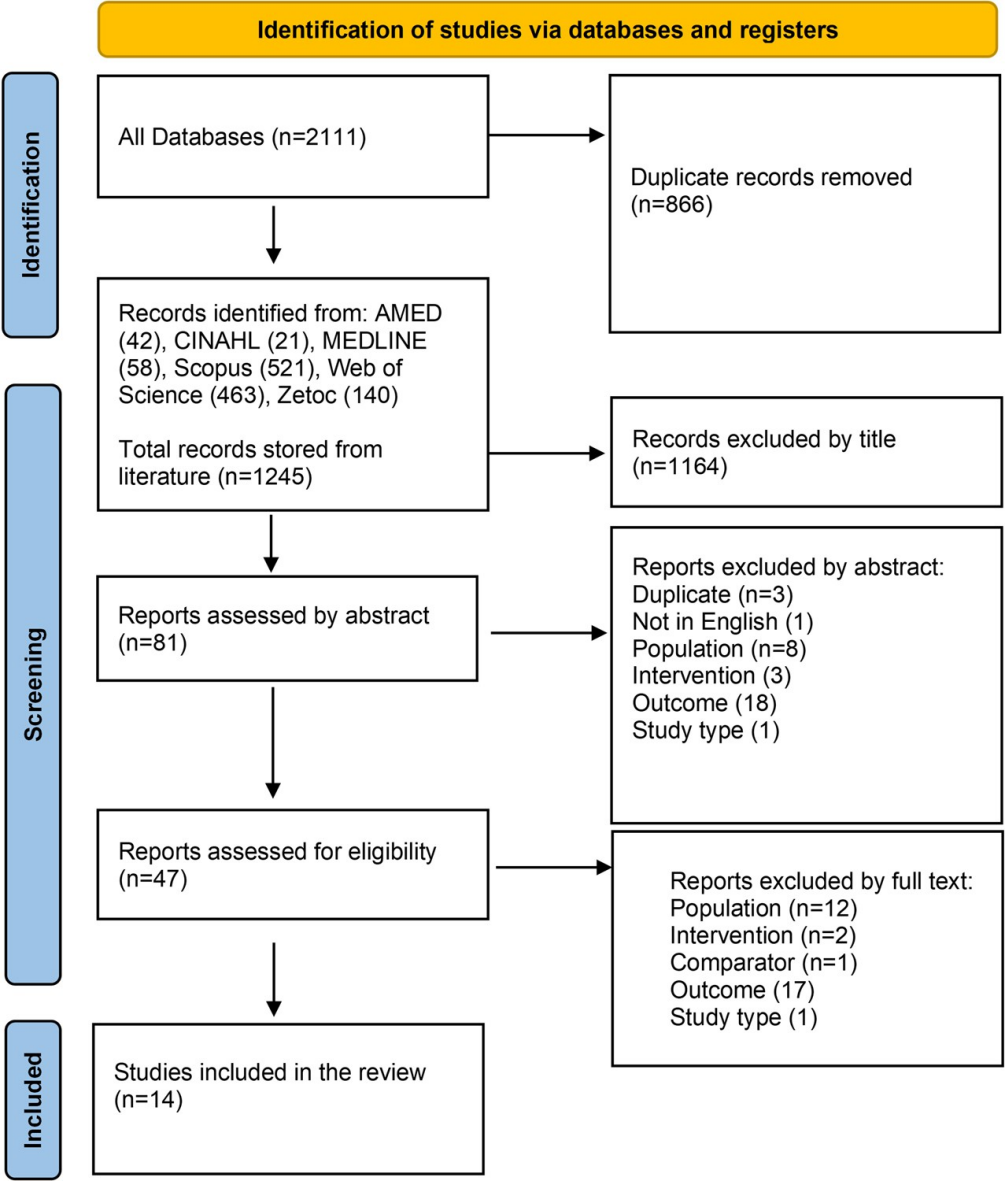
PRISMA flow chart.

From 2111 total citations, 47 full texts were assessed for eligibility. The agreement between reviewers was 100% in all stages. Finally, 14 studies are included in the systematic review. The selection process is illustrated via the PRISMA flow diagram (Fig 1). The reasons for the exclusion of articles are reported in depth for each article in S3 and S4 Files. A full list of the 1245 articles screened is provided in S6 File.

### Study characteristics

#### Article information

Ten articles were RCTs [30–39], one was reported as preliminary RCT [40], and three articles were referred to as randomized trials [5, 41, 42]. The 14 studies were published between 1999 and 2023 and were sourced from different countries: six articles from Asia [30, 33, 34, 37–39], four articles from Europe [31, 32, 35, 36] and four from Australia [5, 40–42].

#### Participant information

Table 1 reports the characteristics of the studies. Seven studies did not report a power calculation [30, 33, 35, 36, 39, 40, 42], whereas three based the power calculation on a measure of disability [31, 32, 38], two on subjective reports of pain [34, 37] and two on EMG data [5, 41]. Only three studies included a comparable number of men and women [34, 37, 40], one did not declare the gender of participants [33], whilst the rest of the studies enrolled only women. Regarding the age of participants, 13 articles enrolled patients aged from 25 to 50 years whereas only one article included participants up to 60 years old [39]. Ten articles recruited participants from the general population, and four recruited from the workplace [30, 32, 35, 36].

#### Exercise interventions

Table 2 describes the exercises performed in each study including a description of the duration, frequency, and intensity of each exercise. A wide range of interventions were considered: functional postural exercises, shoulder stabilization exercises, strength/endurance exercises, CCF training, mixed training, stretching and Feldenkrais, lateral arm raise, suspension training and global postural re-education, and a program of neck specific exercises. Three studies [5, 41, 42] examined comparable interventions: “endurance/strength training” and “low load CCF training”. Two studies [31, 32] used the same neck specific exercise program described by Jull in 2008 [43].

#### Comparators

Regarding the eleven RCTs, three studies [34, 37, 38] offered the control group education regarding ergonomic advice and posture, six [30, 31, 32, 36, 39, 40] did not provide a treatment to the control group, in one [35] they received weekly e-mails about general health education and one [33] provided physical agents.

#### Outcome measures

Each study assessed features of neuromuscular function in addition to pain and disability. With respect to the neuromuscular adaptations investigated, seven studies used EMG to assess neck muscle activity during performance of the CCF test (CCFT) or during maximal voluntary contractions (MVC) of the neck muscles. No study used outcomes from transcranial magnetic stimulation. Other outcomes can be seen in Table 3. Regarding patient reported outcomes, neck pain intensity was assessed in eight studies [30, 31, 33, 34, 36, 37, 39, 40] using the Visual Analog Scale (VAS), whereas five [5, 32, 35, 41, 42] used a NRS, and one study [38] used the Neck Pain and Disability Scale (NPAD). Thirteen studies investigated perceived disability: ten studies [5, 30–33, 37, 39, 40–42] utilized the Neck Disability Index (NDI), one [38] the NPAD and one [36] used the Northwick Park pain questionnaire.

#### Risk of bias

Two articles showed low risk of bias in all domains [34, 35]. Blinding of participants was not discussed as for the type of studies examined it would have shown 100% high risk. Overall, 11% of bias domains were considered “unclear risk”, whereas 10% were considered “high risk”. Specifically, four articles did not provide a control group [5, 32, 41, 42] (Domain 1: Bias arising from the randomization process), one study showed imbalance between groups [31] (Domain 1), and another one had low sample size [38] (Domain 1). Two articles did not provide clear treatment dose [33, 36] (Domain 2: Bias due to deviations from intended interventions). Four articles [30, 36, 37, 40] did not mention blinding of assessment, so their detection bias (Domain 4: Bias in measurement of the outcome) was reported as unclear. Concerning adherence, two studies [36, 39] had a high risk of bias for incomplete outcome data (Domain 3: Bias due to missing outcome data). One article showed poor methodology that affected randomization, measurement of the outcome and reported result [36] (Domain 1, Domain 4: Bias in measurement of the outcome; and Domain 5: Bias in selection of the reported result). A summary of the risk of bias is presented in Table 4.

**Table 4:** Risk of bias.

Study	Domain 1: Bias Arising from the Randomization Process	Domain 2: Bias Due to Deviations from Intended Interventions	Domain 3: Bias Due to Missing Outcome Data	Domain 4: Bias in Measurement of the Outcome	Domain 5: Bias in Selection of the Reported Result	Overall Risk of Bias
**Beer 2012**	Low	Low	Low	Some concerns	Low	High
**Borisut 2013**	Low	Low	Low	Some concerns	Low	High
**Falla 2006**	High	Low	Low	Low	Low	High
**Falla 2008**	Some concerns	Low	Low	Low	Low	High
**Falla 2013**	High	Low	Low	Low	Low	High
**Ghaderi 2017**	Low	Some concerns	Low	Low	Low	High
**Javdaneh 2020**	Low	Low	Low	Low	Low	Low
**Jull 2009**	High	Low	Low	Low	Low	High
**Lidegaard 2013**	Low	Low	Low	Low	Low	Low
**Lundblad 1999**	Some concerns	Some concerns	High	Some concerns	Some concerns	High
**Ma 2011**	Low	Low	Low	Some concerns	Low	High
**Mehri 2020**	High	Low	Low	Low	Low	High
**Mendes-Fernandes 2023**	High	Low	Low	Low	Low	High
**Yan 2022**	Low	Low	High	Low	Low	High

### Results of individual studies and synthesis of results

An overview of the outcome measures and summary results is reported in Table 1 whereas the means, standard deviations and standardized mean difference for each outcome measure can be found in Table 3. Below we provide a summary of changes in EMG measures as these were common to several studies. All other outcomes are summarized in Table 3.

GRADE has been rated for each study. We then grouped the studies per method to provide a general GRADE score for each treatment type evaluated within this systematic review. A summary of the GRADE rating is presented in Table 5.

**Table 5:** GRADE rating of the overall certainty of evidence.

Treatment	Study ID	Risk of Bias	Inconsistency	Indirectness	Imprecision	Other considerations	GRADE rating by study	GRADE rating by treatment
**Postural Exercise**	**Beer 2012**	Serious	Not serious	Not serious	Very Serious	Sample size <24	Very low	Very low
**Resistance Exercises**	**Borisut 2013**	Serious	Not serious	Not serious	Not Serious	None	Moderate	Moderate*
	**Falla 2006**	Serious	Not serious	Not serious	Not Serious	None	Moderate	
	**Falla 2008**	Serious	Not serious	Not serious	Not Serious	None	Moderate	
	**Jull 2009**	Serious	Not serious	Not serious	Not Serious	Sample size <24	Low	
	**Mehri 2020**	Serious	Not serious	Not serious	Serious	None	Moderate	
**CCF training**	**Borisut 2013**	Serious	Not serious	Not serious	Not Serious	None	Moderate	Moderate*
	**Falla 2006**	Serious	Not serious	Not serious	Not Serious	None	Moderate	
	**Falla 2008**	Serious	Not serious	Not serious	Not Serious	None	Moderate	
	**Jull 2009**	Serious	Not serious	Not serious	Not Serious	Sample size <24	Low	
	**Ghaderi 2017**	Serious	Not serious	Not serious	Not Serious	Sample size <24	Low	
**CCF training + Resistance training**	**Borisut 2013**	Serious	Not serious	Not serious	Not Serious	None	Moderate	Moderate
**Mixed physiotherapy exercises**	**Lundblad 1999**	Serious	Not serious	Not serious	Very Serious	None	Very Low	Very Low
**Strength training: shoulder lateral raise**	**Lidegaard 2013**	Not serious	Not serious	Not serious	Serious	None	Moderate	Moderate
**Feldenkrais**	**Lundblad 1999**	Serious	Not serious	Not serious	Very Serious	None	Very Low	Very Low
**Cognitive functional therapy + scapular exercises**	**Javdaneh 2020**	Not serious	Not serious	Not serious	Serious	Publication Bias strongly suspected	Low	Low
**Scapular exercises**	**Javdaneh 2020**	Not serious	Not serious	Not serious	Serious	Publication Bias strongly suspected	Low	Low
**Stretching + neck/shoulder strength training**	**Ma 2011**	Serious	Not serious	Not serious	Very Serious	Sample size <24	Very Low	Very Low
**Suspension exercise for neck**	**Yan 2022**	Serious	Not serious	Not serious	Serious	Sample size <24	Very Low	Very Low
**Neck specific exercise program**	**Falla 2013**	Serious	Not serious	Not serious	Not Serious	Sample size <24	Low	Moderate*
	**Mendes-Fernandes 2023**	Not serious	Not serious	Not serious	Not Serious	None	High	
**Global Postural Re-education**	**Mendes-Fernandes 2023**	Not serious	Not serious	Not serious	Not Serious	None	High	High

Trials with less than 24 subjects have been rated down by 1.

* Indicates total subject count for this treatment is greater than 24

CCFT, cranio-cervical flexion test; GRADE, grading of recommendations, assessment, development and evaluation; ROB, risk of bias; RCT, randomised controlled trial.

### Effectiveness and dosage of different exercise interventions

#### Postural exercises

Postural exercises were investigated in one trial with low risk of bias [40]. They found less activity of the SCM during performance of the CCFT following training, but no effect on pain and disability. Training was performed every day for two weeks with each session lasting 15–20 minutes. Based on the GRADE assessment, there is a very low level of confidence in the evidence supporting the positive outcomes of neuromuscular adaptations associated with postural exercises.

#### Resistance exercises (strength/endurance training)

Resistance exercises were investigated in five trials with low risk of bias [5, 30, 38, 41]. Falla 2006 [41] showed decreased EMG amplitude for SCM and AS during isometric neck flexion contractions at 10, 25 and 50% of MVC post training. Falla 2008 [42] found no differences in EMG amplitude of the SCM during the performance of a repetitive upper limb task where patients were asked “to dot pencil marks in three circles in a clockwise direction” [42]. Jull 2009 [5] found no difference in SCM EMG amplitude during performance of the CCFT following a resistance training intervention.

Borisut 2013 [30] found less EMG amplitude during the performance of MVCs in shoulder elevation (upper trapezius, UT), head raising in a prone position (cervical erector spinae, CE) and neck flexion in a supine position (SCM and anterior scalene, AS). Mehri 2020 [38] showed significantly less EMG amplitude and an early activation onset of the SCM and levator scapulae during the performance of MVCs in neck rotation and neck extension, both performed in sitting. All five articles reported positive effects on pain and disability.

These studies provided 8 to 12 weeks of training with three to seven days of training per week. Repetitions were between 12 to 15 and sets from 1 to 3. Based on the GRADE assessment, there is a moderate level of confidence in the evidence supporting these positive outcomes associated with resistance exercises.

#### CCF training

CCF training was investigated in six trials with serious risk of bias [5, 30, 33, 41]. The assessment of the risk of bias is influenced by the fact that some articles were not an RCT.

Jull 2009 [5] found a significant decrease of SCM EMG amplitude during performance of the CCFT post training. Falla 2006 [41] showed no difference in EMG amplitude for either the SCM or AS during isometric neck flexion contractions at 10, 25 and 50% of MVC. Falla 2008 [42] found no difference in SCM EMG during the repetitive upper limb task described above; Borisut 2013 [30] found less EMG amplitude for UT, CE, SCM and AS during shoulder elevation (testing UT), head raising in prone position (testing CE) and neck flexion in supine position (testing SCM and AS). Ghaderi 2017 [33] found decreased activation of the SCM and splenius capitis (SCap) and AS during performance of the CCFT. All studies reported positive effects on pain and disability.

These studies provided either a 12-, 8- or 6-week trial with one or two sessions of training per day. Repetitions were up to 15 from levels 22 to 30 mmHg on the CCFT. Based on GRADE assessment, there is a moderate level of confidence in the evidence supporting the positive outcomes associated with CCF training.

#### CCF training + strength training

CCF training combined with strength training was investigated in one trial with low risk of bias [30]. Borisut 2013 [30] found less EMG amplitude in UT, CE, SCM and AS during shoulder elevation (testing UT), head raising in prone position (testing CE) and neck flexion in supine position (testing SCM and AS) post training.

This study provided 12-week trial with one session of training per day. Repetitions were up to 15 from levels 22 to 30 mmHg on the CCFT. Based on the GRADE assessment, there is a moderate level of confidence in the evidence supporting the positive outcomes associated with CCF training combined with strength training.

#### Mixed physiotherapy exercises (Strength + stretching + stabilization exercises)

Mixed physiotherapy exercises were investigated in one trial with very high risk of bias [36]. No changes were observed for SCap or UT EMG or shoulder peak torque during the performance of MVCs in shoulder flexion. The study showed no significant differences in pain and disability following the 16-week trial which consisted of two days of training per week (50min per session). Strength training repetitions were up to 15.

Based on GRADE assessment, there is a very low level of confidence in the evidence supporting the outcomes associated with mixed physiotherapy exercises.

#### Strength training: Shoulder lateral raise

Strength training via a shoulder lateral raise was investigated in one article with low risk of bias [35]. This study reported a trend of increased activation of UT and SCap during 90° shoulder abduction, which was statistically significant for the SCap. There was a significant reduction in neck pain intensity.

The training lasted 10 weeks, 5 times per week, increasing the resistance band load every two weeks. Based on the GRADE assessment, there is a moderate level of confidence in the evidence supporting the positive outcomes associated with lateral raise strength training.

#### Feldenkrais intervention

Feldenkrais intervention was investigated by one study with very high risk of bias [36]. They showed less activity of UT, SCM and ES during the performance of MVCs in shoulder flexion. Moreover, a significant reduction in pain and disability was recorded. The training lasted 16 weeks with a total of 16 training of 50 minutes.

Based on GRADE assessment, there is a very low level of confidence in the evidence supporting the positive outcomes associated with Feldenkrais intervention.

#### Cognitive functional therapy + scapular exercises

Cognitive functional therapy combined with scapular exercises was investigated by one study with low risk of bias [34]. They showed a significant increase of activation in trapezius (upper, lower and middle) and serratus anterior during 90° shoulder abduction. There was a significant reduction in neck pain intensity.

The training was provided for 10 weeks, 3 times per week with progressive load, with participants performing 3 sets of 15 repetitions. It should be noted that the authors provided a cognitive functional therapy based on biomechanical concepts that differs from the one described in literature [44]. Additionally, the recruitment period ended in March 2020, and the study was published in March 2020. There are therefore some concerns about publication bias.

Based on GRADE assessment, there is a low level of confidence in the evidence supporting the positive outcomes associated with cognitive functional therapy combined with scapular exercises.

#### Scapular exercises

Scapular exercises were investigated by one article with low risk of bias [34]. They showed a significant increase of activation in trapezius and serratus anterior during 90° shoulder abduction and there was a significant reduction in neck pain intensity.

The training was provided for 10 weeks, 3 times per week with progressive load, 3 sets per 15 repetitions. Based on GRADE assessment, there is a low level of confidence in the evidence supporting the positive outcomes associated with scapular exercises.

#### Stretching + neck/shoulder strength training

Stretching combined with neck/shoulder strength training were investigated by one study with low risk of bias [37]. They showed no differences for both CE and UT during 20 minutes of typing. However, neck pain and disability significantly reduced.

The training, using thera-band, was performed at home for no longer than 20 min 4 times per day for 6 weeks. Based on GRADE assessment, there is a very low level of confidence in the evidence supporting the outcomes associated with these exercises.

#### Suspension exercises

Suspension exercises for the neck were investigated in one study with low risk of bias [39]. They showed a significant reduction of SCM and UT EMG amplitude during performance of the CCFT. Pain and disability also reduced significantly.

The training was provided for 4 weeks, with 3 sessions per week that last for 20/30 minutes. Based on GRADE assessment, there is a very low level of confidence in the evidence supporting the positive outcomes associated with suspension exercises.

#### Neck specific exercise program

A program of neck specific exercises (including CCF training and other low load exercises progressed to higher load resistance training) were investigated in two studies with low risk of bias [31, 32]. Falla 2013 [31] reported less SCM and SCap activity during the performance of isometric contractions for neck flexion, extension and right/left flexion in a sitting position. Both studies reported positive effects on pain and disability. Mendes Fernandes 2023 [32] showed a significant reduction of SCM and AS EMG amplitude during performance of the CCFT. Pain and disability also reduced significantly.

The training was provided for 8 weeks, with 2 sessions per week. Based on GRADE assessment, there is a moderate level of confidence in the evidence supporting the positive outcomes associated with neck specific exercises.

#### Global postural re-education

Global postural re-education was investigated in one study with low risk of bias [32]. They showed a significant reduction of SCM and AS EMG during perfor37nce of the CCFT. Pain and disability also reduced significantly.

The training was provided for 8 weeks, with 2 sessions per week. Based on the GRADE assessment, there is a high level of confidence in the evidence supporting the positive outcomes associated with global postural re-education.

### Meta-analysis

**Fig 2:**
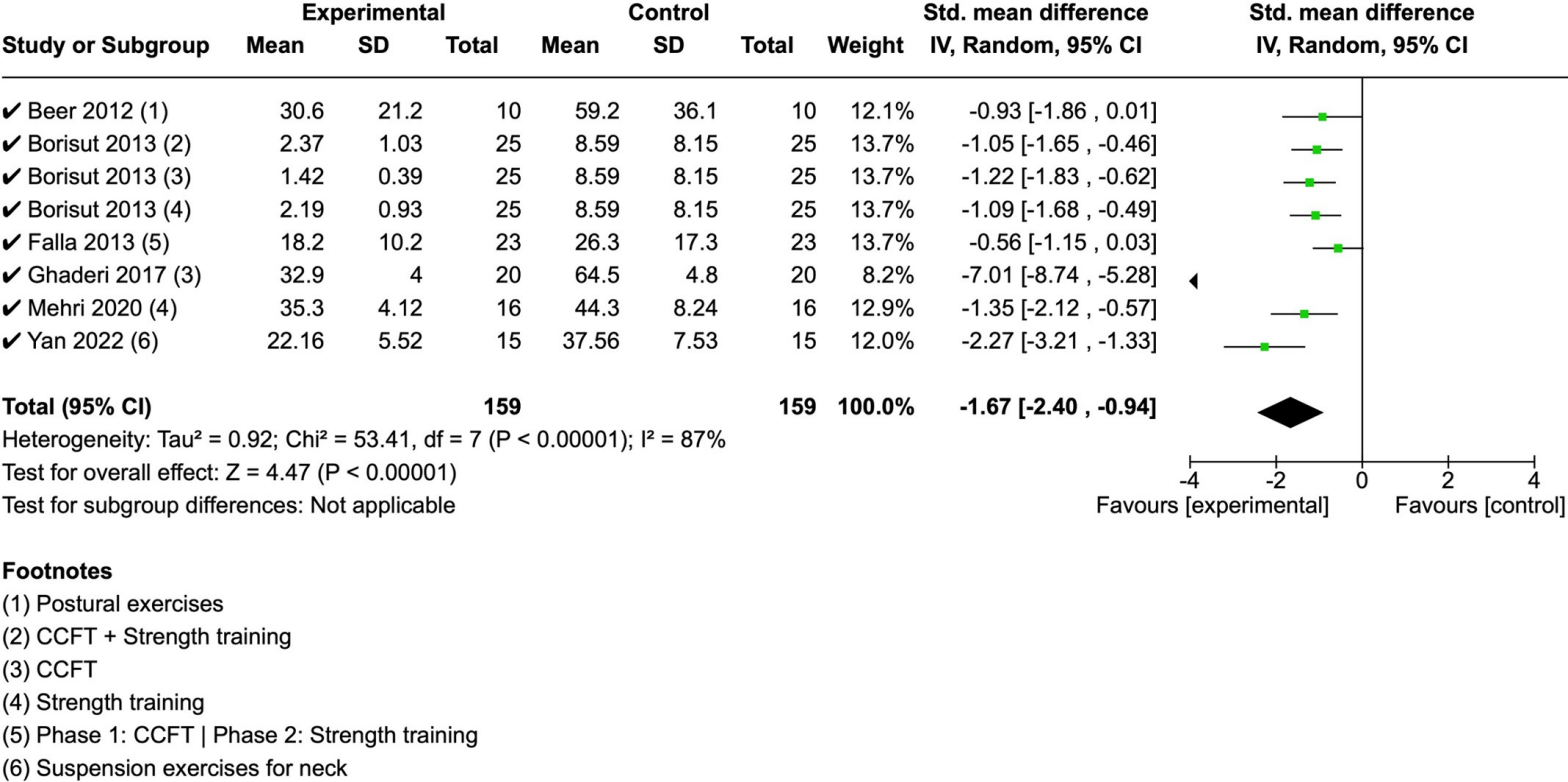
Forest plot showing an estimated overall effect size in favour of the decrease of sternocleidomastoid EMG amplitude due to exercise compared to control interventions.

A meta-analysis could be conducted on SCM EMG amplitude as it was the most common measure investigated. ReviewManager (RevMan) was used to conduct the statistical analysis [26]. Continuous data have been analysed through an inverse variance using a random effect model with 95% confidence interval. The meta-analysis revealed an estimated overall effect size in favour of the decrease of SCM EMG amplitude due to exercise compared to control groups (Fig 2; the data used to generate this figure are provided in S7 File). Moreover, the estimated overall confidence interval does not intersect the “no effect” line, for this reason we can state that the result is statistically significant.

The effect size estimate for the overall outcome is -1.67, with a standard error of 0.73. The Z score is 4.47, indicating statistical significance at a two-tailed significance level < 0.00001. The 95% confidence interval ranges from -2.40 to -0.94, suggesting a statistically significant effect. Overall, this supports a reduction in SCM EMG amplitude in response to training.

## Discussion

This is the first systematic review that comprehensively investigates the effect of exercise on neuromuscular adaptations within the neck muscles in people with chronic non-specific neck pain. Seventeen different exercise approaches from fourteen studies were assessed. Analysis of information was limited by low sample size, differences in exercise dosage, different duration of exercise, and a lack of data about dosage and description of the exercises performed. Thus, a narrative synthesis of the data was carried out for the most part apart from a meta-analysis on changes in SCM EMG amplitude after exercise which shows a reduction of SCM activity after exercise in people with chronic non-specific neck pain. Although this systematic review considered measures obtained with transcranial magnetic stimulation as an outcome measure, none of the 14 articles included used this outcome measure.

All RCTs but two [34, 35] assessed SCM activity via EMG, therefore we were able to synthesize the results from these RCTs [30, 31, 33, 38–40] with a meta-analysis. The overall effect size shows a decrease in SCM EMG activation in response to exercise. Five articles specifically assessed neck muscle activity via EMG as participants performed the CCFT [5, 32, 33, 39, 40] while the other articles examined different tasks. Overall, the results support a reduction of superficial muscle activity (SCM, AS, SCap and UT) during the CCFT following a period of exercise [32, 33, 39, 40]. The study by Jull 2009 [5] was the only article that assessed the deep cervical flexors (longus capitis and longus colli) during performance of the CCFT following exercise and demonstrated an increase in EMG amplitude of the deep cervical flexors during performance of the CCFT following CCF training. The same study also reported no change for the same outcome after neck strength training.

Results varied for other tasks but what became evident from the results is that the type of adaptation closely reflects the type of training performed. For example, Jull 2009 [5] reported improved neuromuscular control of the deep and superficial neck flexors following 6-weeks of CCF training but not following strength training of the neck flexors, even though neck pain intensity reduced by a comparable amount in both groups. Likewise, Falla 2006 [41] reported a reduction of EMG amplitude for the SCM and AS during isometric neck flexion contractions at 10, 25 and 50% of MVC following a resistance training program for the neck flexors yet there were no changes on this task for those that performed low-load CCF training. Furthermore, Falla 2008 [42] found no differences in the EMG amplitude of neck muscles during an upper limb task after either a neck endurance-strength training program or CCF training and Ma 2011 [37] found no differences in the activity of neck muscles during an upper limb task after neck muscle strength training. A further example is the study by Jull 2009 [5] which reported no statistically significant difference in the relative latency of neck muscle activity during rapid arm movements (flexion and extension) following either CCF training or resistance training. However, there were some situations where changes in neuromuscular function did not relate specifically to the type of training performed. For example, Mehri 2020 [38] showed a faster onset of superficial neck muscles (UT, SCM and ES) during an anterior-posterior perturbations following a program of progressive resistance exercises, while Ghaderi 2017 [33] showed a faster onset of SCM AS and SCap during rapid arm movements following CCF training. Thus, the majority of studies from this systematic review suggest that changes in EMG measures (muscle and task) closely match the type of exercise performed although, there were exceptions.

In the current systematic review, we considered studies with a treatment duration of at least 2 weeks. Two studies, Beer 2012 [40] and Yan 2022 [39], provided 2- and 4-weeks of training respectively, whereas Lundblad 1999 [36] used a program of 16 weeks, and the other studies used an exercise program which ranged between 6 to 12 weeks. Both Beer 2012 [40] and Yan 2022 [39] showed that 2 weeks of neck flexor and extensor training are sufficient to decrease SCM and UT EMG during performance of the CCFT. Thus, it appears that cervical neuromuscular adaptations occur rapidly with training, and this is line with an abundance of research reporting early neural adaptations to training [45]. Nevertheless, the advantages of longer periods of training versus shorter durations remain to be investigated.

All articles in this systematic review demonstrated a positive improvement in pain and disability following training, except for the studies by Lundblad 1999 [36] and Beer 2012 [40]. Lundblad 1999 [36] showed no changes in disability in both groups (Feldenkrais or physiotherapy) after 16-weeks of training. However, it should be noted that there were several limitations to this study as detailed in the risk of bias assessment, but additionally, the choice of the outcome measure might have potentially impacted the results. While all other articles used the NDI or NPAD, Lundblad 1999 [36] assessed disability with the Nordic Council of Ministers questionnaire that seems to have suffered floor effects in this situation, since the baseline data were already low.

The study by Beer 2012 [40] was the only study that reported changes in EMG measures of neck muscle activity, but no improvement in either pain or disability. However, the study by Beer 2012 [40] was the one with only a 2-week training program, and as such it was possibly insufficient to result in significant pain relief despite early neural adaptations to training. Further studies are necessary to understand the temporal development of neuromuscular adaptations to training and changes in patient symptoms.

### Strengths and limitations

This review employed rigorous methodology and was conducted according to a published protocol on PROSPERO (CRD42021298831) and reported in line with PRISMA guidance. The quality of the included studies was reduced due to low sample size, differences in exercise dosage, different duration of exercise, and a lack of information on the actual exercises. Moreover, we encountered some difficulties in comparing EMG data between studies given the range of tasks assessed (e.g. CCFT, typing task, shoulder MVC, upper limb task). A further limitation of this systematic review is the language restrictions which were imposed on our searches.

## Conclusions

There is moderate certainty of evidence which supports the use of CCF training (in isolation or in combination resistance training) in patients with chronic non-specific neck pain to induce neural adaptations within the neck muscles. The majority of the results support the notion that neural adaptations to training are specific to the task trained. Further RCTs are needed to evaluate neuromuscular adaptations within deeper neck muscles in response to both CCF training and strength training.

## Supporting information

S1 File Prisma checklist. (DOCX)

S2 File Ovid search strategy. (DOCX)

S3 File Articles excluded by abstract. (DOCX)

S4 File Articles excluded by full text. (DOCX)

S5 File Articles included. (DOCX)

S6 File Full list of articles screened. (XLSX)

S7 File Date used to generate Fig 2. (XLSX)
