# Supplementary material for: The effects of exercise on neuromuscular function in people with chronic neck pain: A systematic review and meta-analysis
Source: PLoS One. 2024 Dec 19;19(12):e0315817. doi: 10.1371/journal.pone.0315817 (PMC11658605; doi:10.1371/journal.pone.0315817)
Supplement: S2 File — (DOCX) [file pone.0315817.s002.docx]

**Supplementary file 2- Ovid search strategy**

Problem [TOTAL 173,460 results]

1. "Neck Pain"[Mesh] 7,793 results
2. Neck AND (pain* OR ache OR myalgia OR stiffness OR soreness OR tenderness OR arthralgia OR strain OR injury OR dysfunction) 117,623 results
3. Cervical AND (pain* OR ache OR myalgia OR stiffness OR soreness OR tenderness OR arthralgia OR Strain OR Injury OR Dysfunction) 173,380 results
4. Neckache* 26 results
5. Cervicalgia* 177 results
6. Cervicodynia* 20 results
7. #1 OR #2 OR #3 OR #4 OR #5 OR #6 173,460 results

Intervention:

1. "Physical Therapy Modalities"[Mesh] 166,737 results
2. “Exercise Therapy” [Mesh] 57,556 results
3. “Exercise” [Mesh] 222,283 results
4. "Muscle Stretching Exercises"[Mesh] 1,919 results
5. Physical AND (Therap* OR Activit*) 874,637 results
6. Physiotherap* 62,650 results
7. Exercise* 456,743 results
8. Training 2,190,361 results
9. Rehabilitation* 487,506 results
10. Remedial Exercise* 1,047 results
11. Stretching 80,870 results
12. Proprioception 42,696 results
13. Proprioceptive 42,696 results
14. #8 OR #9 OR #10 OR #11 OR #12 OR #13 OR #14 OR #15 OR #16 OR #17 OR #18 OR #19 OR #20 3,611,761 results

Outcome:

1. “Motor Skills” [Mesh] 25,642 results
2. “Muscle Strength” [Mesh] 39,611 results
3. “Muscle fibers, skeletal” [Mesh] OR “Muscle fibers, Fast-Twitch” [Mesh] OR “Muscle fibers, Slow-Twitch” [Mesh] 6,485 results
4. “Muscle spindles” [Mesh] 3,590 results
5. Muscle AND (architecture OR size OR fibre* OR volume OR timing OR activity OR performance OR strength OR damage* OR activation OR contraction OR spindle*) 674,665 results
6. Motor unit* AND (recruitment OR firing OR excitability OR firing rate OR behaviour OR discharge rate* OR Skill*) 76,117 results
7. Anatomical cross-section area 240 results
8. Physiological cross-Section area 197 results
9. Moment arm OR Movement arms 28,239 results
10. Neuromuscular 97,924 results
11. (Motor OR sensorimotor) AND cortex 66,890 results
12. (Sensorimotor OR Cortex OR Motor OR Spinal) AND change* 231,011 results
13. Firing frequenc* 15,582 results
14. Myotendinous stiffness 52 results
15. Strength (Maximal strength, Maximum strength, Explosive Strength) 415,060 results
16. Power 433,611 results
17. Maximal voluntary contraction 7,353 results
18. Co-activaction 588,972 results
19. Coactivation 27,548 results
20. Myofibrillar mechanisms 1,061 results

Tools:

1. “Muscle Strength Dynamometer” [Mesh] OR Dynamometer 8,595 results
2. “Magnetic Resonance Imaging” [Mesh] OR Magnetic Resonance Imaging 615,871 results
3. Interpolated Twitch Technique OR twitch interpolation technique 326 results
4. Electromyography 92,287 results
5. #22 OR #23 OR #24 OR #25 OR #26 OR #27 OR #28 OR #29 OR #30 OR #31 OR #32 OR #33 OR #34 OR #35 OR #36 OR #37 OR 38 OR #39 OR #40 OR #41 2,860,522 results
6. #7 AND #21 AND #46 10,797 results
