# Supplementary material for: The effects of exercise on neuromuscular function in people with chronic neck pain: A systematic review and meta-analysis
Source: PLoS One. 2024 Dec 19;19(12):e0315817. doi: 10.1371/journal.pone.0315817 (PMC11658605; doi:10.1371/journal.pone.0315817)
Supplement: S3 File — (DOCX) [file pone.0315817.s003.docx]

| **Reason** | **Articles** |
| --- | --- |
| **Duplicate** (3) | Ang 2009, Bjorn 2009, Jull 2009 |
| **Non-English** (1) | Arami 2012 |
| **Population**  Specific neck pain (3) | França 2008, Karthi 2019, O’Leary 2012 |
| **Population** Shoulder pain (4) | Andersen 2014, Hagberg 2000, Saeterbakken 2020, Saeterbakken 2017 |
| **Population** Healty people (1) | Kramer 2013 |
| **Intervention**  no exercise treatment (2) | Bahat 2018, Khosrokiani 2021 |
| **Intervention**  no neck treatment (1) | Chiu 2005 |
| **Outcome**  no emg assessment (18) | Akhter 2014, Ashfaq 2021, Bobos 2016, Evans 2012, Fatima 2022, Gallego Izquierdo 2016, Groeneweg 2017, Iversen 2018, Jordan 1998, Karlsson 2014, Matias 2019, Murray 2017, Murray 2015, Pérez-Cabezas 2020, Randløv 1998, Wang 2018, Willaert 2020, Ylinen 2007 |
| **Study type**  Secondary analysis (1) | Falla 2012 |

1. *Akhter S, Khan M, Ali SS, Soomro RR. Role of manual therapy with exercise regime versus exercise regime alone in the management of non-specific chronic neck pain. Pak J Pharm Sci. 2014 Nov;27(6 Suppl):2125-8.*
2. *Andersen CH, Andersen LL, Zebis MK, Sjøgaard G. Effect of scapular function training on chronic pain in the neck/shoulder region: a randomized controlled trial. J Occup Rehabil. 2014 Jun;24(2):316-24.*
3. *Ang BO, Monnier A, Harms-Ringdahl K. Neck/shoulder exercise for neck pain in air force helicopter pilots: a randomized controlled trial. Spine (Phila Pa 1976). 2009 Jul 15;34(16):E544-51.*
4. *Arami, J., Rezasoltani, A., Khalkhali Zaavieh, M. & Rahnama, L. The effect of two exercise therapy programs (proprioceptive and endurance training) to treat patients with chronic non-specific neck pain. Journal of Babol University of Medical Sciences, 2012 14****,*** *78-84.*
5. *Ashfaq R, Riaz H. Effect of Pressure biofeedback training on deep cervical flexors endurance in patients with mechanical neck pain: A randomized controlled trial. Pak J Med Sci. 2021;37(2):550-5.*
6. *Sarig Bahat H, Croft K, Carter C, Hoddinott A, Sprecher E, Treleaven J. Remote kinematic training for patients with chronic neck pain: a randomised controlled trial. Eur Spine J. 2018 Jun;27(6):1309-23.*
7. *Bjorn O., Monnier A., Harms-Ringdahl K. Neck/shoulder exercise for neck pain in air force helicopter pilots Spine 2009 34(16):E544-E551*
8. *Bobos P, Billis E, Papanikolaou DT, Koutsojannis C, MacDermid JC. Does Deep Cervical Flexor Muscle Training Affect Pain Pressure Thresholds of Myofascial Trigger Points in Patients with Chronic Neck Pain? A Prospective Randomized Controlled Trial. Rehabil Res Pract. 2016;2016:6480826.*
9. *Chiu TT, Hui-Chan CW, Chein G. A randomized clinical trial of TENS and exercise for patients with chronic neck pain. Clin Rehabil. 2005 Dec;19(8):850-60..*
10. *Gallego Izquierdo T, Pecos-Martin D, Lluch Girbés E, Plaza-Manzano G, Rodríguez Caldentey R, Mayor Melús R, et al. Comparison of cranio-cervical flexion training versus cervical proprioception training in patients with chronic neck pain: A randomized controlled clinical trial. J Rehabil Med. 2016 Jan;48(1):48-55.*
11. *Groeneweg R, van Assen L, Kropman H, Leopold H, Mulder J, Smits-Engelsman BCM, et al. Manual therapy compared with physical therapy in patients with non-specific neck pain: a randomized controlled trial. Chiropr Man Therap. 2017;25:12.*
12. *Iversen VM, Vasseljen O, Mork PJ, Fimland MS. Resistance training vs general physical exercise in multidisciplinary rehabilitation of chronic neck pain: A randomized controlled trial. J Rehabil Med. 2018 Aug 22;50(8):743-50.*
13. *Jordan A, Bendix T, Nielsen H, Hansen FR, Høst D, Winkel A. Intensive training, physiotherapy, or manipulation for patients with chronic neck pain. A prospective, single-blinded, randomized clinical trial. Spine (Phila Pa 1976). 1998 Feb 1;23(3):311-8; discussion 319.*
14. *Jull GA, Falla D, Vicenzino B, Hodges PW. The effect of therapeutic exercise on activation of the deep cervical flexor muscles in people with chronic neck pain. Man Ther. 2009 Dec;14(6):696-701.*
15. *Evans R, Bronfort G, Schulz C, Maiers M, Bracha Y, Svendsen K, et al. Supervised exercise with and without spinal manipulation performs similarly and better than home exercise for chronic neck pain: a randomized controlled trial. Spine (Phila Pa 1976). 2012 May 15;37(11):903-14.*
16. *Fatima A, Veqar Z, Zaidi S, Tanwar T. Effects of scapular stabilization and upper limb proprioception as an adjunct to cervical stabilization in chronic neck pain patients: A randomized controlled trial. J Bodyw Mov Ther. 2022 Jan;29:291-301.*
17. *Falla D, O'Leary S, Farina D, Jull G. The change in deep cervical flexor activity after training is associated with the degree of pain reduction in patients with chronic neck pain. Clin J Pain. 2012 Sep;28(7):628-34.*
18. *França DL, Senna-Fernandes V, Cortez CM, Jackson MN, Bernardo-Filho M, Guimarães MA. Tension neck syndrome treated by acupuncture combined with physiotherapy: a comparative clinical trial (pilot study). Complement Ther Med. 2008 Oct;16(5):268-77.*
19. *Hagberg M, Harms-Ringdahl K, Nisell R, Hjelm EW. Rehabilitation of neck-shoulder pain in women industrial workers: a randomized trial comparing isometric shoulder endurance training with isometric shoulder strength training. Arch Phys Med Rehabil. 2000 Aug;81(8):1051-8.*
20. *Karlsson L, Takala EP, Gerdle B, Larsson B. Evaluation of pain and function after two home exercise programs in a clinical trial on women with chronic neck pain - with special emphasises on completers and responders. BMC Musculoskelet Disord. 2014 Jan 8;15:6.*
21. *Karthi, M., Gopalswami, A. D. & Aseer, A. L. 2019. Efficacy Of Endurance Training On Deep Cervical Flexor Muscles Using Pressure Feedback In Mechanical Neck Pain. International Journal of Physiotherapy, 6****,*** *95-101.*
22. *Kramer M, Hohl K, Bockholt U, Schneider F, Dehner C. Training effects of combined resistance and proprioceptive neck muscle exercising. J Back Musculoskelet Rehabil. 2013;26(2):189-97.*
23. *Khosrokiani Z, Letafatkar A, Gladin A. Lumbar motor control training as a complementary treatment for chronic neck pain: A randomized controlled trial. Clin Rehabil. 2022 Jan;36(1):99-112.*
24. *Matias, B. A., Vieira, I., Pereira, A., Duarte, M. & Silva, A. G. 2019. Pain neuroscience education plus exercise compared with exercise in university students with chronic idiopathic neck pain. International Journal of Therapy and Rehabilitation, 26.*
25. *Murray M, Lange B, Nørnberg BR, Søgaard K, Sjøgaard G. Self-administered physical exercise training as treatment of neck and shoulder pain among military helicopter pilots and crew: a randomized controlled trial. BMC Musculoskelet Disord. 2017 Apr 7;18(1):147.*
26. *Murray M, Lange B, Nørnberg BR, Søgaard K, Sjøgaard G. Specific exercise training for reducing neck and shoulder pain among military helicopter pilots and crew members: a randomized controlled trial protocol. BMC Musculoskelet Disord. 2015 Aug 19;16:198.*
27. *O'Leary S, Jull G, Kim M, Uthaikhup S, Vicenzino B. Training mode-dependent changes in motor performance in neck pain. Arch Phys Med Rehabil. 2012 Jul;93(7):1225-33.*
28. *Saeterbakken AH, Makrygiannis P, Stien N, Solstad TEJ, Shaw M, Andersen V, et al. Dose-response of resistance training for neck-and shoulder pain relief: a workplace intervention study. BMC Sports Sci Med Rehabil. 2020;12:8.*
29. *Saeterbakken AH, Nordengen S, Andersen V, Fimland MS. Nordic walking and specific strength training for neck- and shoulder pain in office workers: a pilot-study. Eur J Phys Rehabil Med. 2017 Dec;53(6):928-35.*
30. *Pérez-Cabezas V, Ruiz-Molinero C, Jimenez-Rejano JJ, Chamorro-Moriana G, Gonzalez-Medina G, Chillon-Martinez R. Effectiveness of an Eye-Cervical Re-Education Program in Chronic Neck Pain: A Randomized Clinical Trial. Evid Based Complement Alternat Med. 2020;2020:2760413.*
31. *Randløv A, Ostergaard M, Manniche C, Kryger P, Jordan A, Heegaard S, et al. Intensive dynamic training for females with chronic neck/shoulder pain. A randomized controlled trial. Clin Rehabil. 1998 Jun;12(3):200-10.*
32. *Wang H, Jiang E, Wang K, Deng Z, Zhan H, Shen Z, et al. Shi's Daoyin Therapy for Neck Pain: A Randomized Controlled Trial. Evid Based Complement Alternat Med. 2018;2018:4983891.*
33. *Willaert W, Malfliet A, Coppieters I, Lenoir D, De Pauw R, Danneels L, et al. Does Pain Neuroscience Education and Cognition-Targeted Motor Control Training Improve Cervical Motor Output? Secondary Analysis of a Randomized Clinical Trial. Pain Pract. 2020 Jul;20(6):600-14.*
34. *Ylinen J, Häkkinen A, Nykänen M, Kautiainen H, Takala EP. Neck muscle training in the treatment of chronic neck pain: a three-year follow-up study. Eura Medicophys. 2007 Jun;43(2):161-9.*
