# Supplementary material for: The effects of exercise on neuromuscular function in people with chronic neck pain: A systematic review and meta-analysis
Source: PLoS One. 2024 Dec 19;19(12):e0315817. doi: 10.1371/journal.pone.0315817 (PMC11658605; doi:10.1371/journal.pone.0315817)
Supplement: S4 File — (DOCX) [file pone.0315817.s004.docx]

| **Reason** | **Articles** |
| --- | --- |
| **Population**  Shoulder pain (3) | Alhlgren 2001, Andersen 2008b, Ang 2009 |
| **Population**  Specific neck pain pain (8) | Andersen 2008a, Andersen 2009, Ang 2005, Bahat 2020, Brage 2015, Bronfort 2001, Dellve 2011, Suvarnato 2019 |
| **Population**  healthy people (1) | Hsu 2020 |
| **Intervention**  treatment <2 weeks (1) | LLuch 2014 |
| **Intervention**  No neck treatment (1) | Park 2020 |
| **Comparator**  Alexander technique (1) | Becker 2021 |
| **Outcome** No emg (17) | Abadiyan 2021, Andersen 2008c, Andersen 2011, Avellanet 2021, Bak 2018, Caputo 2017, Churng 2018, Gallego Izquerdo 2016, Hakkinen 2008, Iqbal 2021, Kuo 2020, Lee 2016, Nezamuddin 2013, O’Leary 2007, Rajalaxmi 2019, Rajalaxmi 2020, Raju 2019 |
| **Study type**  Study protocol (1) | Mendes-Fernandes 2021 |

1. *Abadiyan F, Hadadnezhad M, Khosrokiani Z, Letafatkar A, Akhshik H. Adding a smartphone app to global postural re-education to improve neck pain, posture, quality of life, and endurance in people with nonspecific neck pain: a randomized controlled trial. Trials. 2021 Apr 12;22(1):274.*
2. *Ahlgren C., Waling K., Kadi F. Djupsjöbacka M. Effects on physical performance and pain from three dynamic training programs for women with work-related trapezius myalgia J Rehabil Med 2001 33:162-169*
3. *Andersen LL, Andersen CH, Zebis MK, Nielsen PK, Søgaard K, Sjøgaard G. Effect of physical training on function of chronically painful muscles: a randomized controlled trial. J Appl Physiol (1985). 2008a Dec;105(6):1796-801.*
4. *Andersen LL, Jørgensen MB, Blangsted AK, Pedersen MT, Hansen EA, Sjøgaard G. A randomized controlled intervention trial to relieve and prevent neck/shoulder pain. Med Sci Sports Exerc. 2008b Jun;40(6):983-90.*
5. *Andersen LL, Kjaer M, Søgaard K, Hansen L, Kryger AI, Sjøgaard G. Effect of two contrasting types of physical exercise on chronic neck muscle pain. Arthritis Rheum. 2008c Jan 15;59(1):84-91.*
6. *Andersen LL, Andersen JL, Suetta C, Kjaer M, Søgaard K, Sjøgaard G. Effect of contrasting physical exercise interventions on rapid force capacity of chronically painful muscles. J Appl Physiol (1985). 2009 Nov;107(5):1413-9.*
7. *Andersen LL, Saervoll CA, Mortensen OS, Poulsen OM, Hannerz H, Zebis MK. Effectiveness of small daily amounts of progressive resistance training for frequent neck/shoulder pain: randomised controlled trial. Pain. 2011 Feb;152(2):440-6.*
8. *Ang B, Linder J, Harms-Ringdahl K. Neck strength and myoelectric fatigue in fighter and helicopter pilots with a history of neck pain. Aviat Space Environ Med. 2005 Apr;76(4):375-80.*
9. *Ang B. O., Monnier A., Harms-Ringdahl K. “Neck/shoulder exercise for neck pain in air force helicopter pilots” Spine 2009 34(16):E544-E551*
10. *Avellanet M, Boada-Pladellorens A, Pages E, Dorca A, Sabria B, Pfeifer M, et al. A Comparative Study of a Novel Postural Garment Versus Exercise for Women with Nonspecific Cervical Pain: A Randomized Cross-over Trial. Spine (Phila Pa 1976). 2021 Nov 15;46(22):1517-24.*
11. *Bahat HS, German D, Palomo G, Gold H, Nir YF. Self-Kinematic Training for Flight-Associated Neck Pain: a Randomized Controlled Trial. Aerosp Med Hum Perform. 2020 Oct 1;91(10):790-7.*
12. *Bak Y. Effects of neck exercise program on a taxi driver’s with chronic neck pain International Journal of Advanced Nursing Education and Research 2018 2:13-18*
13. *Becker J. J., McIsaac T. L., Copeland S. L., Cohen R. G. Alexander technique vs. targeted exercise for neck pain – a preliminary comparison” Appl. Sci. 2021 11:4640*
14. *Brage K., Ris I., Falla D., Søgaard K., Juul-Kristensen B. Pain education combined with neck- and aerobic training is more effective at relieving chronic neck pain than pain education alone - A preliminary randomized controlled trial Man Ther. 2015 20:686-693*
15. *Bronfort G, Evans R, Nelson B, Aker PD, Goldsmith CH, Vernon H. A randomized clinical trial of exercise and spinal manipulation for patients with chronic neck pain. Spine (Phila Pa 1976). 2001 Apr 1;26(7):788-97; discussion 798-9.*
16. *Caputo GM, Di Bari M, Naranjo Orellana J. Group-based exercise at workplace: short-term effects of neck and shoulder resistance training in video display unit workers with work-related chronic neck pain-a pilot randomized trial. Clin Rheumatol. 2017 Oct;36(10):2325-33.*
17. *Chung S, Jeong YG. Effects of the craniocervical flexion and isometric neck exercise compared in patients with chronic neck pain: A randomized controlled trial. Physiother Theory Pract. 2018 Dec;34(12):916-25.*
18. *Dellve L, Ahlstrom L, Jonsson A, Sandsjö L, Forsman M, Lindegård A, et al. Myofeedback training and intensive muscular strength training to decrease pain and improve work ability among female workers on long-term sick leave with neck pain: a randomized controlled trial. Int Arch Occup Environ Health. 2011 Mar;84(3):335-46.*
19. *Gallego Izquierdo T, Pecos-Martin D, Lluch Girbés E, Plaza-Manzano G, Rodríguez Caldentey R, Mayor Melús R, et al. Comparison of cranio-cervical flexion training versus cervical proprioception training in patients with chronic neck pain: A randomized controlled clinical trial. J Rehabil Med. 2016 Jan;48(1):48-55.*
20. *Häkkinen A, Kautiainen H, Hannonen P, Ylinen J. Strength training and stretching versus stretching only in the treatment of patients with chronic neck pain: a randomized one-year follow-up study. Clin Rehabil. 2008 Jul;22(7):592-600.*
21. *Hsu WL, Chen CP, Nikkhoo M, Lin CF, Ching CT, Niu CC, et al. Fatigue changes neck muscle control and deteriorates postural stability during arm movement perturbations in patients with chronic neck pain. Spine J. 2020 Apr;20(4):530-7.*
22. *Iqbal ZA, Alghadir AH, Anwer S. Efficacy of Deep Cervical Flexor Muscle Training on Neck Pain, Functional Disability, and Muscle Endurance in School Teachers: A Clinical Trial. Biomed Res Int. 2021;2021:7190808.*
23. *Kuo YL, Lee TH, Tsai YJ. Evaluation of a Cervical Stabilization Exercise Program for Pain, Disability, and Physical Impairments in University Violinists with Nonspecific Neck Pain. Int J Environ Res Public Health. 2020 Jul 28;17(15):5430.*
24. *Lee KW, Kim WH. Effect of thoracic manipulation and deep craniocervical flexor training on pain, mobility, strength, and disability of the neck of patients with chronic nonspecific neck pain: a randomized clinical trial. J Phys Ther Sci. 2016 Jan;28(1):175-80.*
25. *Lluch E, Schomacher J, Gizzi L, Petzke F, Seegar D, Falla D. Immediate effects of active cranio-cervical flexion exercise versus passive mobilisation of the upper cervical spine on pain and performance on the cranio-cervical flexion test. Man Ther. 2014 Feb;19(1):25-31.*
26. *Mendes-Fernandes T, Puente-González AS, Márquez-Vera MA, Vila-Chã C, Méndez-Sánchez R. Effects of Global Postural Reeducation versus Specific Therapeutic Neck Exercises on Pain, Disability, Postural Control, and Neuromuscular Efficiency in Women with Chronic Nonspecific Neck Pain: Study Protocol for a Randomized, Parallel, Clinical Trial. Int J Environ Res Public Health. 2021 Oct 12;18(20):10704.*
27. *Nezamuddin M., Khan S. A., Hameed U., Anwer S. Efficacy of pressure biofeedback guided deep cervical flexor training on forward head posture in visual display terminal operators Indian J of Physiotherapy & Occupational Therapy 2013 7(4):141-146*
28. *O'Leary S, Jull G, Kim M, Vicenzino B. Specificity in retraining craniocervical flexor muscle performance. J Orthop Sports Phys Ther. 2007 Jan;37(1):3-9.*
29. *Park SH, Lee MM. Effects of Lower Trapezius Strengthening Exercises on Pain, Dysfunction, Posture Alignment, Muscle Thickness and Contraction Rate in Patients with Neck Pain; Randomized Controlled Trial. Med Sci Monit. 2020 Mar 23;26:e920208.*
30. *Rajalaxmi.V., Jibi Paul, M. Manoj Abraham et al. Efficacy of Motor Control and Endurance Exercises in Neck Pain: A Pilot Study. Indian J Forensic Med Pathol. 2019;12(1):19-24.*
31. *Rajalaxmi V., Manickam M., Srilakshmi M., Arunselvi J. The role of multistep core stability exercise with and without conventional neck exercises in the treatment of chronic non-specific neck pain a randomized controlled trial Biomedicine 2020 40(2):232-235*
32. *Raju A. S., Apparao P., Chintada G. S., Chaturbadi P. A comparative study on deep cervical flexors training and neck stabilization exercises in subjects with chronic neck pain Indian Journal of Physiotherapy and Occupational Therapy 2019 13(2)*
33. *Suvarnato T. et al. (2019) “Effect of specific deep cervical muscle exercises on functional disability, pain intensity, craniovertebral angle, and neck-muscle strength in chronic mechanical neck pain: a randomized controlled trial” Journal of Pain Research 12:915-925*
