# Supplementary material for: The effects of exercise on neuromuscular function in people with chronic neck pain: A systematic review and meta-analysis
Source: PLoS One. 2024 Dec 19;19(12):e0315817. doi: 10.1371/journal.pone.0315817 (PMC11658605; doi:10.1371/journal.pone.0315817)
Supplement: S5 File — (DOCX) [file pone.0315817.s005.docx]

**Appendix 5: Articles included**

|  | Author | Date | Title |
| --- | --- | --- | --- |
| 1 | Beer | 2012 | Can a functional postural exercise improve performance in the cranio-cervical flexion test? A preliminary study |
| 2 | Borisut | 2013 | Effects of strength and endurance training of superficial and deep neck muscles on muscle activities and pain levels of females with chronic neck pain |
| 3 | Falla | 2006 | An endurance-strength training regime is effective in reducing myoelectric manifestations of cervical flexor muscle fatigue in females with chronic neck pain |
| 4 | Falla | 2008 | Training the cervical muscles with prescribed motor tasks does not change muscle activation during a functional activity |
| 5 | Falla | 2013 | Effectiveness of an 8-week exercise programme on pain and specificity of neck muscle activity in patients with chronic neck pain: A randomized controlled study |
| 6 | Ghaderi | 2017 | The clinical and EMG assessment of the effects of stabilization exercise on nonspecific chronic neck pain: A randomized controlled trial |
| 7 | Javdaneh | 2020 | Scapular exercise combined with cognitive functional therapy is more effective at reducing chronic neck pain and kinesiophobia than scapular exercise alone: a randomized controlled trial |
| 8 | Jull | 2009 | The effect of therapeutic exercise on activation of the deep cervical flexor muscles in people with chronic neck pain |
| 9 | Lidegaard | 2013 | Effect of Brief Daily Resistance Training on Occupational Neck/Shoulder Muscle Activity in Office Workers with Chronic Pain: Randomized Controlled Trial |
| 10 | Lundblad | 1999 | Randomized controlled trial of physiotherapy and Feldenkrais interventions in female workers with neck-shoulder complaints |
| 11 | Ma | 2011 | Comparing biofeedback with active exercise and passive treatment for the management of work-related neck and shoulder pain: A randomized controlled trial |
| 12 | Mehri | 2020 | Effects of Corrective Exercises on Posture, Pain, and Muscle Activation of Patients With Chronic Neck Pain Exposed to Anterior-Posterior Perturbation |
| 13 | Mendes Fernandes | 2023 | A randomized controlled trial on the effects of “Global Postural Re-education” *versus* neck specific exercise on pain, disability, postural control, and neuromuscular features in women with chronic non-specific neck pain |
| 14 | Yan | 2022 | Effect of sling exercise therapy on surface electromyography and muscle thickness of superficial cervical muscle groups in female patients with chronic neck pain |
